# Comparative phylogeography of amphibians and reptiles in Algeria suggests common causes for the east-west phylogeographic breaks in the Maghreb

**DOI:** 10.1371/journal.pone.0201218

**Published:** 2018-08-29

**Authors:** Menad Beddek, Saliha Zenboudji-Beddek, Philippe Geniez, Raouaa Fathalla, Patricia Sourouille, Véronique Arnal, Boualem Dellaoui, Fatiha Koudache, Salah Telailia, Olivier Peyre, Pierre-André Crochet

**Affiliations:** 1 CEFE, CNRS, Univ. Montpellier, Univ Paul Valéry Montpellier 3, INRA, EPHE, IRD, Montpellier, France; 2 Naturalia Environnement, Site Agroparc, Avignon, France; 3 Unité de recherche de biodiversité et biologie des populations, Faculté des sciences de Tunis, Université de Tunis Elmanar, Tunis, Tunisie; 4 Département de l’Environnement, Faculté des Sciences, Université Djillali Liabes, Sidi Bel Abbes, Algérie; 5 Département des Sciences Agronomiques, Faculté des Sciences de la Nature et de la Vie, Université Chadli Bendjedid, El Tarf, Algérie; State Museum of Natural History, GERMANY

## Abstract

A series of phylogeographic studies in the Maghreb identified a repeated pattern of deep genetic divergence between an eastern (Tunisia) and western (Morocco) lineage for several taxa but lack of sampling in Algeria made it difficult to know if the range limits between the eastern and western lineages were shared among taxa or not. To address this question, we designed a comparative phylogeographic study using 8 reptile and 3 amphibian species with wide distribution in the Maghreb as models. We selected species where previous studies had identified an East-West phylogeographic divide and collected sampled in Algeria to 1) examine whether the simple East-West divergence pattern still holds after filling the sampling gap in Algeria or if more complex diversity patterns emerge; 2) if the E-W pattern still holds, test whether the limits between the E and W clades are shared between species, suggesting that common historical process caused the E-W divergences; 3) if E-W limits are shared between species, use information on the age of the divergence to identify possible geological or climatic events that could have triggered these E-W differentiations. We found that the E-W pattern was generally maintained after additional sampling in Algeria and identified two common disjunction areas, one around the Algeria-Morocco border, the other one in Kabylia (central Algeria), suggesting that common historical mechanisms caused the E-W divergences in the Maghreb. Our estimates for the times to most common recent ancestors to the E and W clades span a wide range between the Messinian salinity crisis and the Plio-Pleistocene limit (except for one older split), suggesting different origins for the initial divergences and subsequent preservation of the E and W lineages in common climatic refugia in the west and the east of the Maghreb.

## Introduction

Understanding how global biodiversity patterns emerged from historical and ecological processes has been a major task of biogeographical studies ever since they started; identifying areas of higher diversity and endemism is also the basis for applied conservation [1,2]. The Mediterranean Basin constitutes a global biodiversity hotspot [3] and as a consequence has been identified as a conservation priority [4] because it contains high rates of endemism and elevated species richness in several taxa: higher plants [3,5], freshwater fishes [6], mammals [7], reptiles and amphibians [8], dragonflies [9].

Within the Mediterranean Basin, the Maghreb (here defined as Northern Algeria, Tunisia, and Morocco) is a well-defined ecological unit surrounded by natural barriers: the Sahara to the south and the east, the Mediterranean to the north and the Atlantic Ocean to the west [10]. It contains several plant diversity hotspots [11,12] and is inhabited by many endemic species of reptiles and amphibians [8,13]. There is however a huge discrepancy between the amount of knowledge on biodiversity in the northern and the southern sides of the Mediterranean basin [14,15] and further research in the Maghreb would allow a better understanding of the historical processes that shaped Mediterranean biodiversity at a regional scale.

Many studies have attempted to understand how the Mediterranean biodiversity hotspot was generated (for ex. [16–20]). One of the emerging patterns of these studies is that for many species North African and South European populations are genetically related, demonstrating that the Mediterranean Sea has not always prevented dispersal and gene flow between Europe and North Africa. Much interchange occurred via land connexion before the opening of Gibraltar (see references in [19]) but in some species dispersal occurred after the filing of the Mediterranean Basin and the opening of Gibraltar at the end of the Messinian crisis (e.g. [21,22]). This succession of divergence on either side of the Mediterranean Sea and colonisation across the Mediterranean contributed building biodiversity in the Mediterranean Basin, and the Maghreb has been the source of colonisation of Europe in many thermophilic organisms [23–25].

Another factor explaining the high diversity of the Mediterranean Basin is its complex topography and history that promoted elevated rates of in situ diversification and allowed persistence of this diversity in multiple refugia during cold periods of the glacial cycles. This phenomenon has been particularly well studied in the north of the region (southern Europe: see [26]) but the Maghreb has not been as intensively investigated. Like southern Europe, the Maghreb encompasses a large diversity of landscapes and bioclimatic regions [27] and a series of phylogeographic studies also identified a high level genetic variability in many species ([25] and references therein). However, we still lack a comprehensive understanding of the main drivers of diversification within the Maghreb.

One of the shortcoming that currently impedes understanding of how the biodiversity patterns in North Africa emerged is the lack of a multi-taxa phylogeography approach. As advocated by [28], a comparative phylogeography approach is needed to identify relevant historical causes explaining observed diversity patterns. Because many concurrent demographic, geological and /or climatic events can shape the current distribution of any given taxon, only “the comparison of geographical patterns of genetic variation among multiple co-distributed taxa” [29] can enlighten demographic and historical processes responsible for the building up of genetic diversity within species and between closely related species [30]. Accordingly, a common genetic diversity pattern shared in a given area between several taxa is supposed to be shaped by the same historical events. For example, location of contact zones between divergent lineages in Europe exhibit common patterns that have been linked to common glacial refugia and post-glacial colonization routes [31–33]. No such studies are available for the Maghreb, but several biodiversity hotspots have been suggested to correspond with glacial refugia [20,25].

While we still lack a proper comparative phylogeography approach in the Maghreb, the existing phylogeographic studies identify a seemingly repeated geographical pattern of deep lineages divergence between Tunisia in the east and Morocco in the west of the region. This E-W divergence is verified in the majority of the studied taxa: reptiles (*Natrix maura*, [34,35], *Chalcides ocellatus*, [36], *Trogonophis wiegmanni* [37], *Ptyodactylus oudrii*, [38,39], *Timon tangitanus* / *pater*, [40,41], amphibians (*Hyla meridionalis*, [42,43], *Pelophylax saharicus*, [44]), scorpions [45], rodents (*Jaculus orientalis*, [46], *Meriones shawii* complex, [47]), molluscs: *Cornu aspersum* [48]. In birds the pattern does not seem to be as general but it has been recovered in Dupont’s Lark [49] and Crested Lark [50] at least.

This common pattern is currently difficult to interpret because in most cases there are few or no sample from Algeria. As a consequence, the locations of the suture zones between the E and W clades are unknown. The reasons for the lack of sampling in Algeria are mainly the current political unrest and safety issues, resulting in a large sampling gap that currently precludes our understanding of the origin of the E-W genetic divide in Maghreban lineages.

In order to improve our knowledge on the origin of current biodiversity patterns in the Maghreb, we designed a comparative phylogeography approach in the Maghreb, specifically aiming at reducing the sampling gap in Algeria. We propose that reptiles and amphibians constitute a relevant model for this study, firstly because previous studies allowed us to select several taxa showing deep E-W genetic divergence, secondly because their relatively lower dispersal ability relative to birds or large mammals make them more sensitive to landscape barriers or climatic oscillations. The aim of this study were thus: 1) to examine whether the simple East-West divergence pattern that divides the populations of many species into two main clades distributed east and west of the Maghreb still holds after filling the sampling gap in Algeria or if more complex diversity patterns with different geographical distributions emerge; 2) if the E-W pattern still holds, to test whether the limits between the E and W clades are shared between species, suggesting that common historical process caused the E-W divergences; 3) if E-W limits are shared between species, to use information on the age of the divergence to identify possible geological or climatic events that could have triggered these E-W differentiations.

To carry out this study, we selected species that fulfil two criteria: 1) they occur throughout most of the Maghreb and 2) they have been the subject of previous phylogeographic studies and exhibit deep divergence between an eastern and western clade. We conducted targeted sampling in Algeria to produce original sequence data that were merged with sequence available in Genbank. We use mitochondrial DNA (mtDNA) as a proxy for evolutionary units because it allowed us to investigate a larger number of species (11, see material and methods) with our available resources. We are fully aware of the risks of single-markers studies [51] and of the benefits of multi-locus approaches but multi-locus analyses would have constrained us to use a reduced number of species. Moreover, most of former studies were mainly or exclusively based on mtDNA, so mtDNA is the only way to merge all existing data together. Even if several evolutionary mechanisms can cause discordance in divergence patterns between mtDNA and the nuclear genome [52], the most common pattern is by far general congruence between the two types of markers [53,54], making us confident that our general conclusion will not be affected by this problem.

## Material and methods

### Ethic statement

Specimens from Morocco were collected under the authorisation n°15 HCEFLCD / DLCDPN / DPRN / CFF delivered by the « Haut-Commissariat aux Eaux et Forêts et à la Lutte Contre la Désertification ». The list of protected species of reptiles and amphibians in Algeria is has been determined by the Executive Decree n°12–235 05/24/2012. Among the species we have used, the following are protected under Algerian law: *Acanthodactylus savignyi*, *A*. *blanci*, *Chalcides ocellatus*, *Timon pater*, *T*. *tangitanus*. However, the legislative authority supposed to deliver capture licenses for protected species according to the Law n°14–07 relative to Natural Resources is not identified in any official document and Algerian biologists therefore have to collect tissue samples or voucher specimens without legal permits. Most tissue samples used for this study have been obtained by non-invasive processes (tail tips or muscle samples from road-killed specimens). Voucher collection has followed practice of Algerian herpetologists. Field work in protected areas has been approved, and often supported in the field, by local authorities (see list in Acknowledgments).

### Sampling

A total of 323 tissue samples (mainly muscle samples, sometimes buccal swabs) mostly collected from the field in Northern Algeria from 2013 to 2015 and deposited in the CEFE-CNRS tissues collection in Montpellier (BEV collection) were used in this study (S1 Table). The samples belong to 11 taxa, 8 reptiles and 3 amphibians. All these taxa had been subjected to previous phylogeographic studies revealing an E-W divergence in mtDNA: *Acanthodactylus erythrurus* [3], *Chalcides ocellatus* [36], *Hemorrhois hippocrepis* [55], *Natrix maura* [34], *Podarcis vaucheri* [56–58], *Ptyodactylus oudrii*, [38,39], *Timon pater / tangitanus* [40,41]. The samples origin and their GenBank accession codes are detailed in S1 Table.

### DNA extraction, amplification and sequencing

DNA was extracted with the Qiagen DNeasy Blood and Tissues kit following the manufacturer’s instructions. Standard protocols were used for Polymerase Chain Reaction (PCR) amplifications with the Sigma kit. The choice of the molecular marker sequenced for each species was dependant on previous published studies for this species. We used sequences of ND4 and adjacent tRNA for *Acanthodactylus erythrurus*, *Podarcis vaucheri* and *Natrix maura*, CytB for *Chalcides ocellatus*, *Hemorrhois hippocrepis* and *Discoglossus pictus*, CO1 for *Hyla meridionalis* and *Pelophylax saharicus*, 16S RNA for *Timon pater* / *tangitanus* and 12S RNA for *Ptyodactylus oudrii*, allowing us to merge our sequences with the sequences already published. PCR conditions and primers are detailed in S2 Table. Purification and sequencing of PCR products were carried out by Eurofins MWG Germany. The chromatograms were checked using Codon code aligner version 4.2.7 for Windows (CodonCode Corporation, Dedham, MA, USA). The alignment of the sequences was carried out with KlustalW implemented in Mega version 7.0 [59] and verified by eye.

### Phylogenetic analyses

Rooted trees were produced with two approaches. Firstly, RaxML [60] with partitioning and the online platform PhyML [61] with automatic AIC criterion based substitution model selection and NNI option for tree improvement were used for maximum likelihood reconstructions. The support of the nodes was evaluated by bootstrapping with 1000 replicates. As GTR is the only nucleotide substitution model available in RAxML, GTR + I + G was applied to all partitions. Secondly, MrBayes 3.2 [62] was used for Bayesian inference (BI). Two separate runs of 6 million generations were conducted with a sampling every 500 trees. Four Monte Carlo Markov chains were initiated for which the starting topology was randomly selected. The convergence of the runs was visualized using Tracer 1.4 [63]. The first 10% trees were eliminated as the burn-in period. The majority consensus tree was computed from the remaining trees. For BI methods, best evolution models and partitioning were calculated using Partitionfinder [64]. Fifty-six different models were tested and the Bayesian information criterion (BIC) was used for the final choice of model. Both reconstruction methods gave similar topologies for all taxa except *Timon* and we retained the BI topology for inference and mapping of the deepest lineages (see below). For *Timon* we did not manage to obtain the same topology with and without our short sequences so we inferred the topology using long sequences from Genbank only and placed the new Algerian lineage (only represented by our short sequences) in this tree in a second step. For *P*. *oudrii* and *T*. *wiegmanni*, we added longer Genbank sequences to the main alignment comprising our sequences (12 S for *P*. *oudrii*, 16 S for *T*. *wiegmanni*, see S1 Table) to improve the nodal robustness.

### Time calibration

The time to most recent common ancestor (TMRCA) of the eastern and western mitochondrial clades were estimated as follows: time calibrated trees were obtained with Beast v1.8.2 [65] under an uncorrelated lognormal relaxed clock; we used a Yule prior on rates of cladogenesis for almost all taxa since the trees included outgroup species but the constant size prior was used for *P*. *saharicus*; four runs were conducted for each taxon (parameters of chain’s lengths and substitution rates are given in Table 1). For each time calibration tree, we used published substitution rates: [66] for *A*. *erythrurus*, medium evolution rate (see [67]) for *H*. *meridionalis* and *D*. *pictus*, [39] for *P*. *oudrii*, [68] for *P*. *vaucheri*, [69] for *T*. *pater* and *T*. *tangitanus*, [70] for *P*. *saharicus*, except *N*. *maura* for which we used published time calibrated nodes [71]. The four runs were first analysed in Tracer 1.4 [63] to check the convergence of the chains and then combined in LogCombiner v1.8.2 [72]. TreeAnnotator v1.7.0 [73] was used to produce the chronograms. We performed the dating analysis only on 8 of the 11 taxa (see Table 1). For *C*. *ocellatus* the analysis was already done by [36]. For *H*. *hippocrepis* and *T*. *wiegmanni* because of non-convergence of the runs we did not manage to have accurate results even after several runs (>4).

**Table 1:** Substitution models, tree prior, mean substitution rate and the number of Monte Carlo Markov chains used in the reconstruction of time calibrated trees.

Taxon	Substitution Model	Tree prior	Mean Rate	MCMC
***Acanthodactylus erythrurus***	GTR	Yule process	0.0174	2.5X10^7^
***Natrix maura***	TN93+G+I	Yule process	-	2X10^7^
***Podarcis vaucheri***	TN93+G+I	Yule process	0.0174	2X10^7^
***Ptyodactylus oudrii***	GTR+G	Yule process	0.00755	2X10^7^
***Timon spp***	GTR	Yule process	0.0016	2X10^7^
***Discoglossus pictus***	TN93+G+I	Yule process	0.00825	2X10^7^
***Hyla meridionalis***	TN93+G+I	Yule process	0.0085	2X10^7^
***Pelophylax saharicus***	HKY	Constant size	0.0195	2X10^7^

### Lineage delimitation and genetic diversity

To estimate genetic diversity for each species we used Dnasp [74] to compute the following indices: S: number of segregating sites, H: number of haplotypes, Hd: haplotypic diversity. To assess if an E-W divide is the main process shaping mitochondrial diversity in our taxa, we used the deepest split in the phylogeny of every taxon to identify the two main clades and mapped the distribution of these two clades. To explore further significant structure in mitochondrial diversity, we used the automatic barcode gap discovery (ABGD) method [75]. Although this program is used for species delimitation, we use it to objectively describe distinct evolutionary units regardless to their taxonomic rank. The ABGD approach is based on the principle that divergence between individuals belonging to the same “species” (here lineage) is smaller than divergence between individuals from different “species”. This approach is designed to work with short DNA sequences, hence it is appropriate for our short sequence data. The analyses were run with default settings. However, as recommended by the program’s author when the run with the default gap width gives only one partition, the relative gap width (X = 1.5 by default) was decreased to 1.2 for *Trogonophis wiegmanni* and to 1.0 for *Acanthodactylus erythrurus*. The most stable distribution value of the initial partition was used to decide the most likely number of distinct lineages. We spatially displayed the results using ARC map10 to get an explicit map of lineage distribution, then, the geographical limits between linages were plotted using the mid distance between the nearest individuals of adjacent lineages.

## Results

### Mitochondrial diversity and geographical distribution of major clades

**Fig 1:**
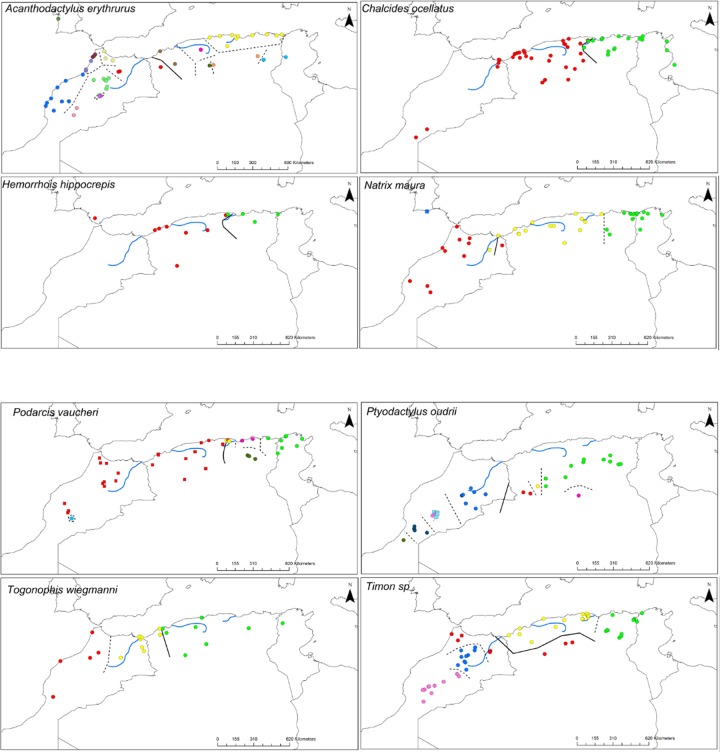
Geographic distribution of the reptiles’ genetic lineages delimited by ABGD program. The thick black lines correspond to the suture zones of the deepest splits for each taxon. Dashed lines correspond to the geographical limits between lineages delimited by ABGD program. The ABGD lineages are represented by the coloured dots.

**Fig 2:**
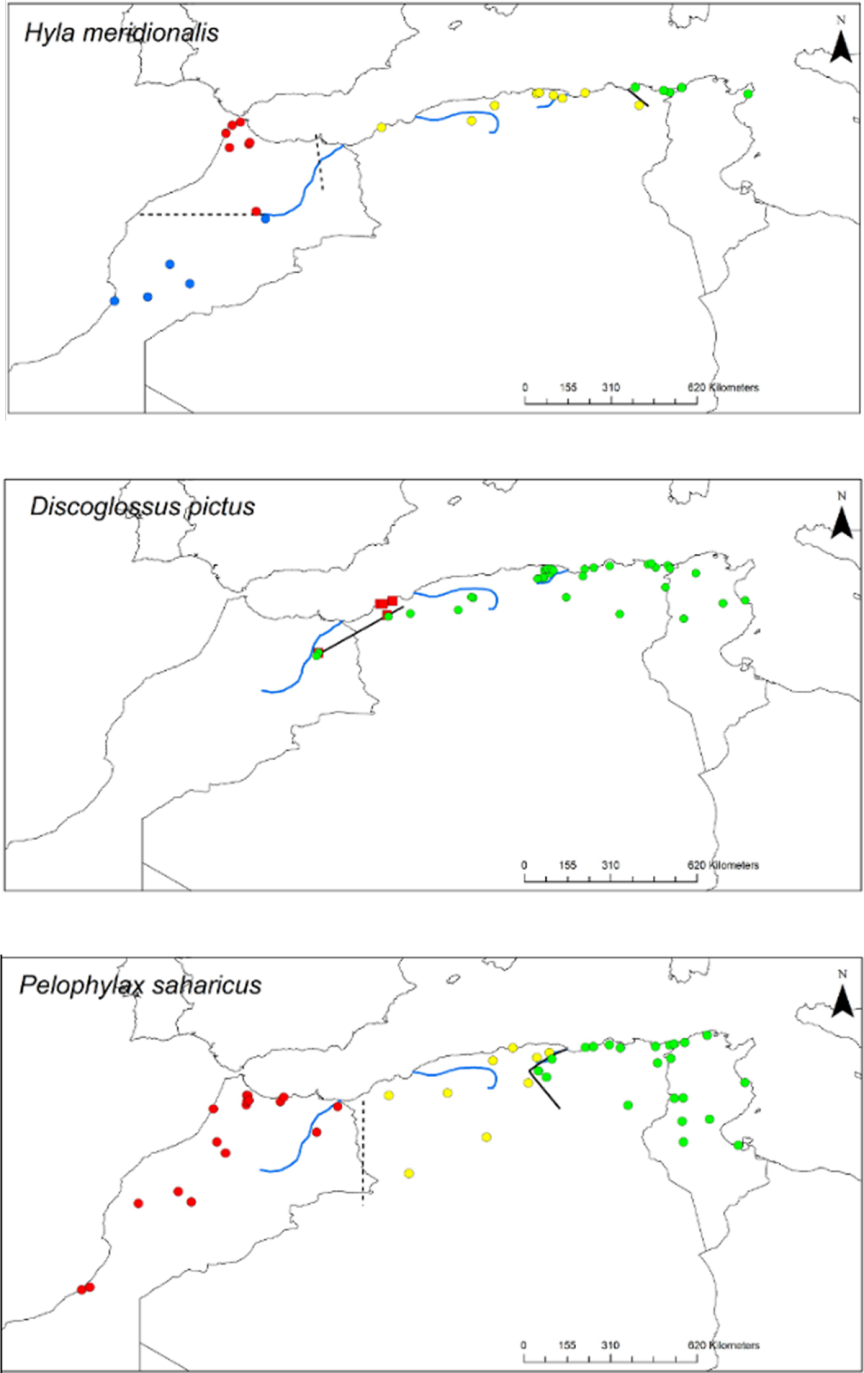
Geographic distribution of the amphibians’ genetic lineages delimited by ABGD program. The thick black lines correspond to the suture zones of the deepest splits for each taxon. Dashed lines correspond to the geographical limits between lineages delimited by ABGD program. The ABGD lineages are represented by the coloured dots.

Delimitation of mtDNA clades by ABGD was concordant with previous studies but our additional sampling in Algeria revealed previously overlooked lineages in several taxa (*Timon spp*, *H*. *meridionalis*, *A*. *erythrurus*, *P*. *oudrii*, *P*. *vaucheri*: compare Figs 1 and 2 with previous studies). Patterns of mitochondrial diversity in Algeria can be grouped into three main categories: i) Algeria is inhabited by the same lineage that is found in Morocco and Tunisia (*H*. *hippocrepis*, *C*. *ocellatus*, *D*. *pictus*), ii) a central lineage occupies parts of Algeria and sometimes adjacent territories between the previously identified eastern and western lineages that occur in Tunisia and Morocco respectively (*Pelophylax*, *Timon*, *Natrix*, *Trogonophis*, *Hyla*) or iii) Algeria is inhabited by several lineages with restricted range (*Podarcis*, *Acanthodactylus*, *Ptyodactylus*). Several of the lineages we have identified are endemic from Algeria based on current sampling (see Figs 1 and 2).

Sequence divergence between clades and diversity within clades are given in Table 2. In spite of the diversity of phylogeographic patterns uncovered by sampling in Algeria, the previously identified general pattern of an E-W divergence corresponding with the major break in mtDNA diversity (deepest node within North Africa) still holds for most taxa (see Figs 3–13).

**Fig 3:**
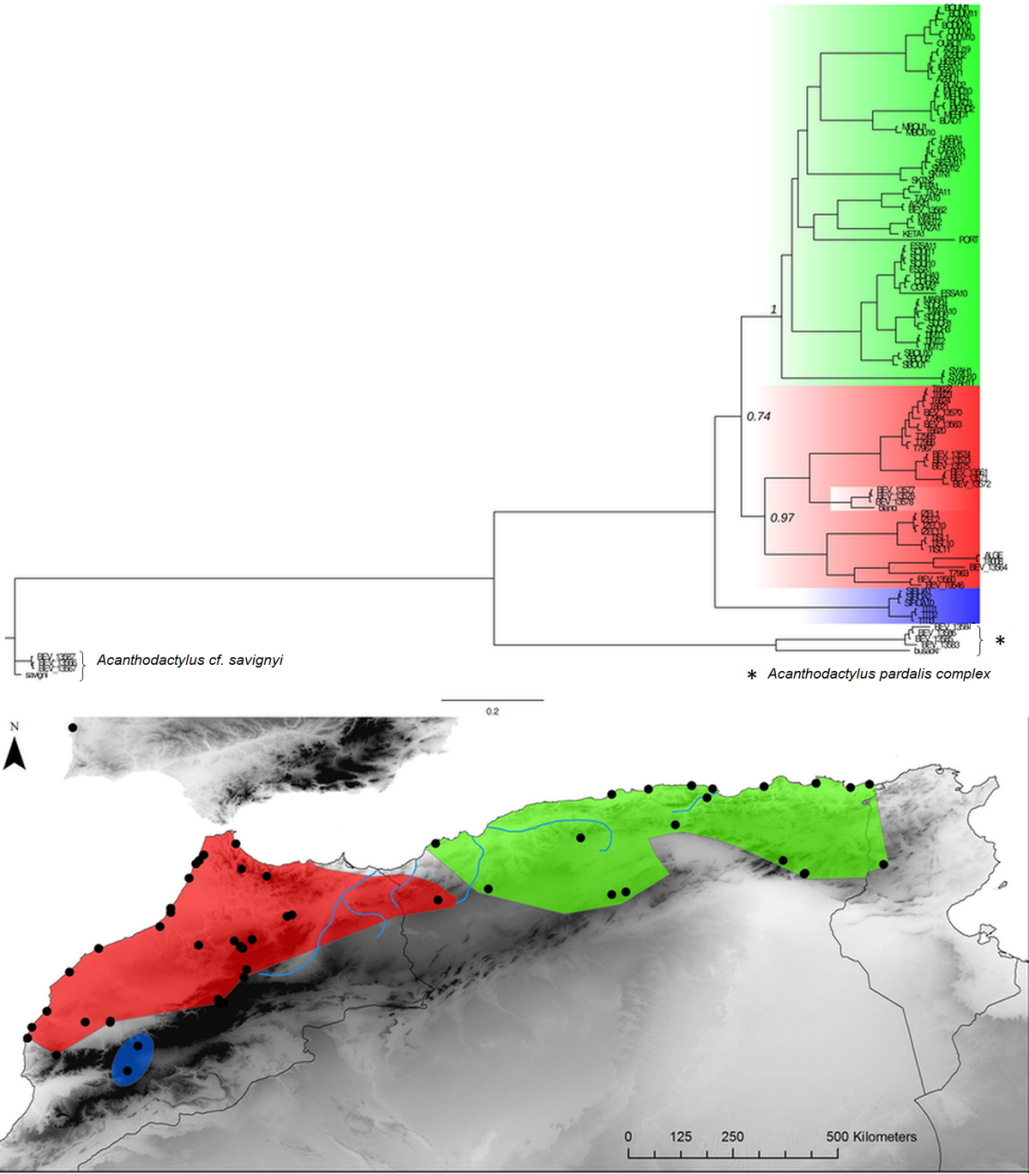
*Acanthodactylus erythrurus* Bayesian inference phylogenetic tree and geographic distribution of deep clades. The green area in the map corresponds to the distribution of the eastern clade populations and the red area corresponds to the distribution of the western clade populations. The blue lineage is missing nodal support, its phylogenetic position is not resolved with the present data set.

**Fig 4:**
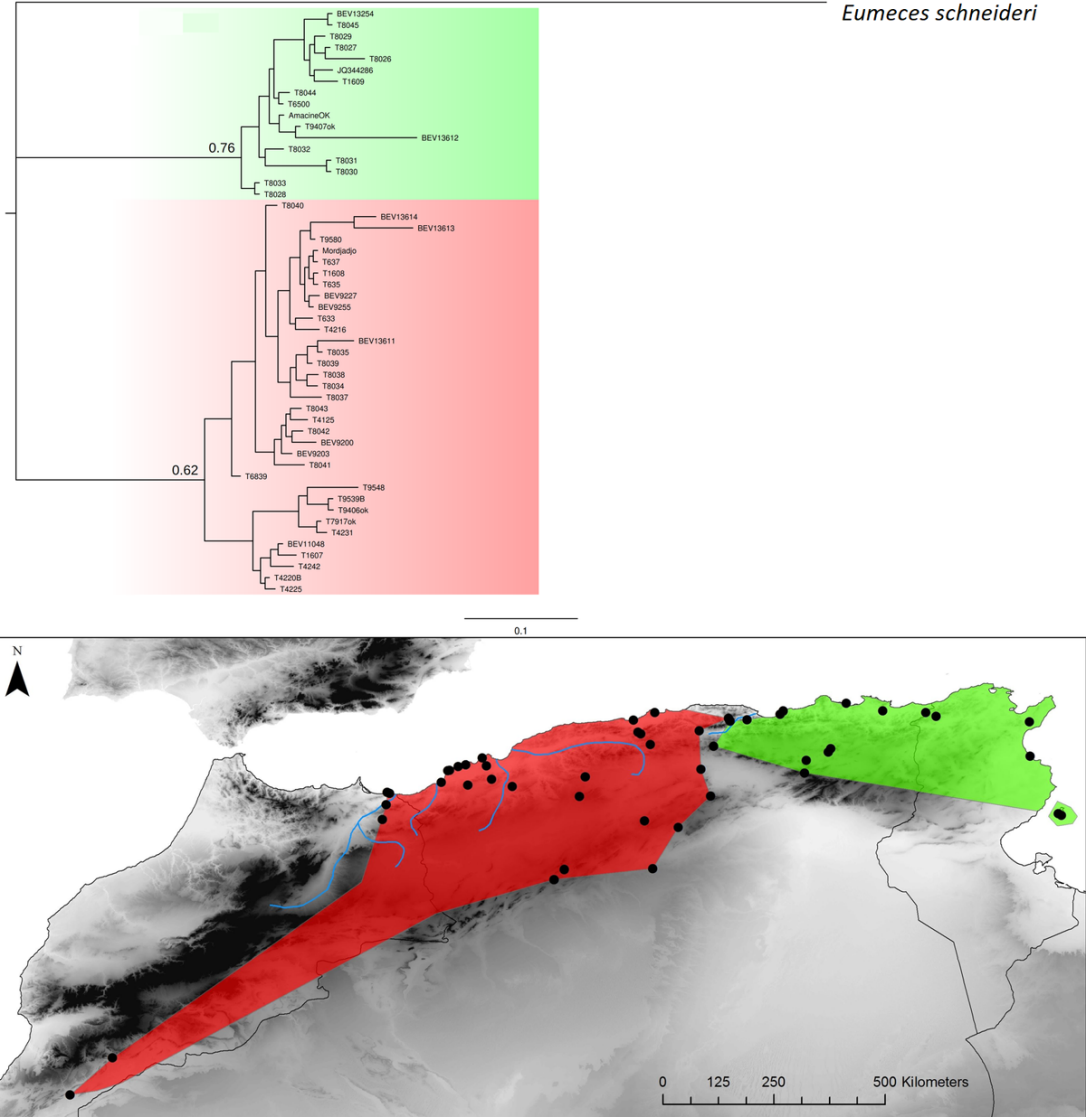
*Chalcides ocellatus* Bayesian inference phylogenetic tree and geographic distribution of deep clades.

**Fig 5:**
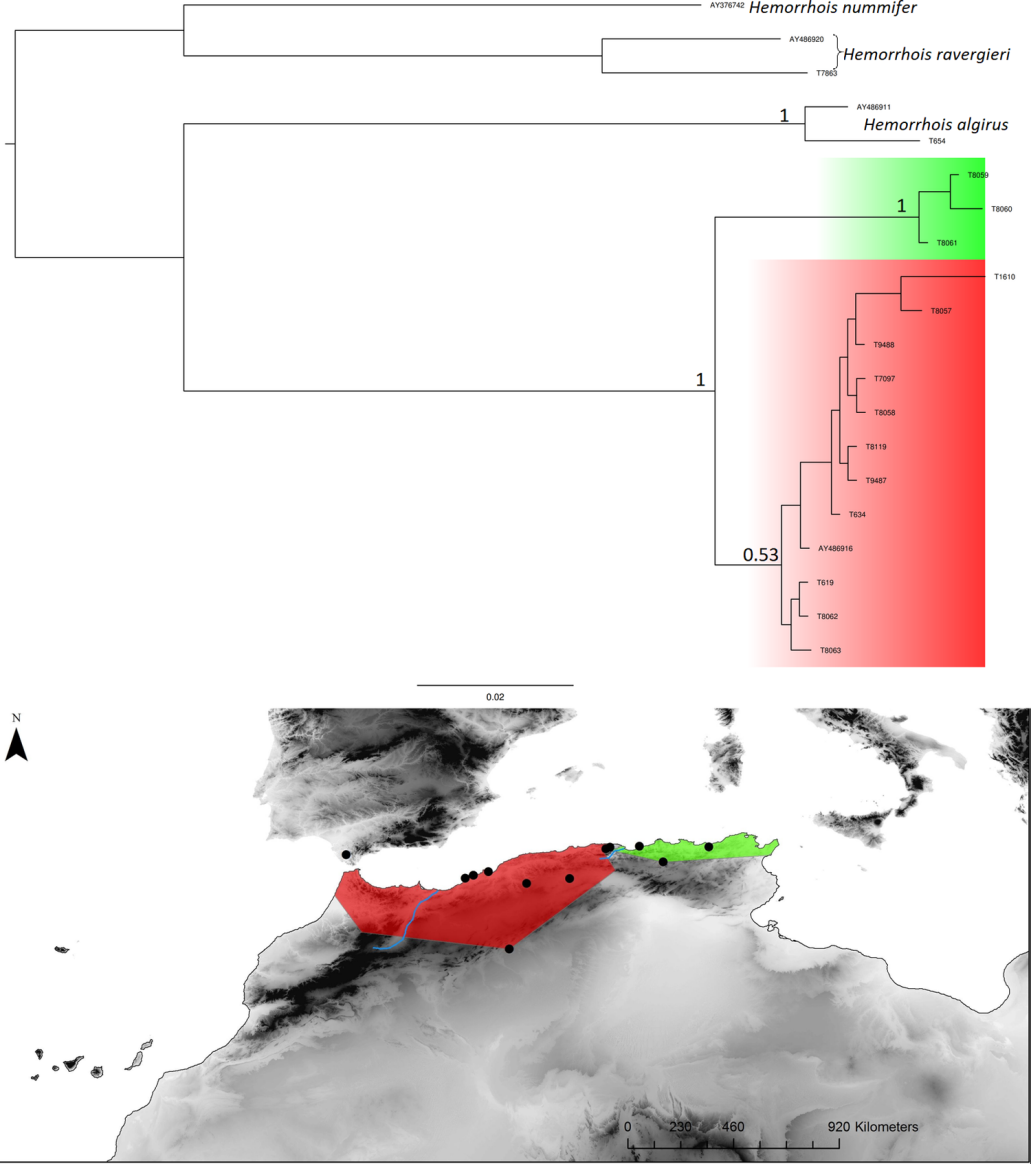
*Hemorrhois hippocrepis* Bayesian inference phylogenetic tree and geographic distribution of deep clades.

**Fig 6:**
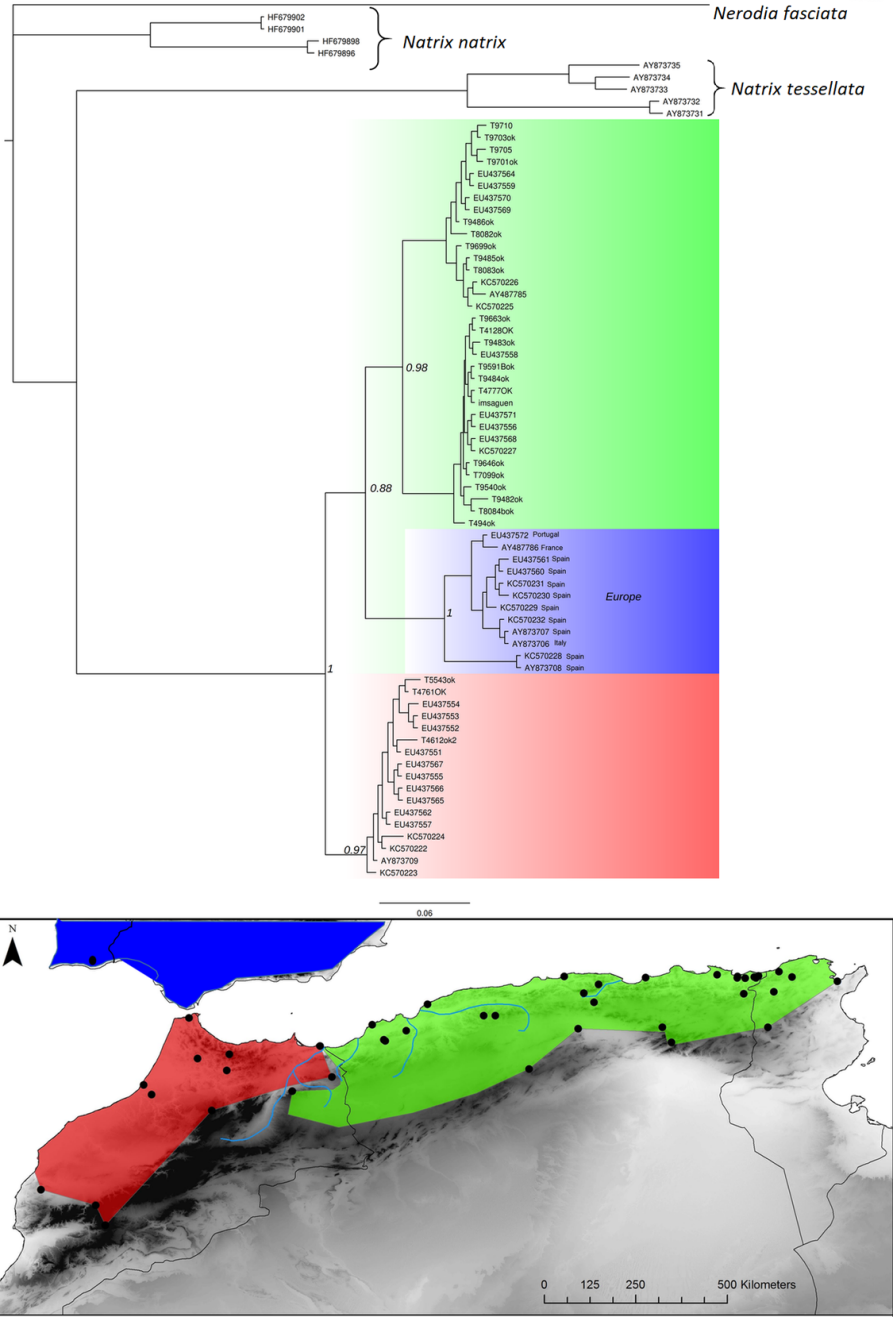
*Natrix maura* Bayesian inference phylogenetic tree and geographic distribution of deep clades.

**Fig 7:**
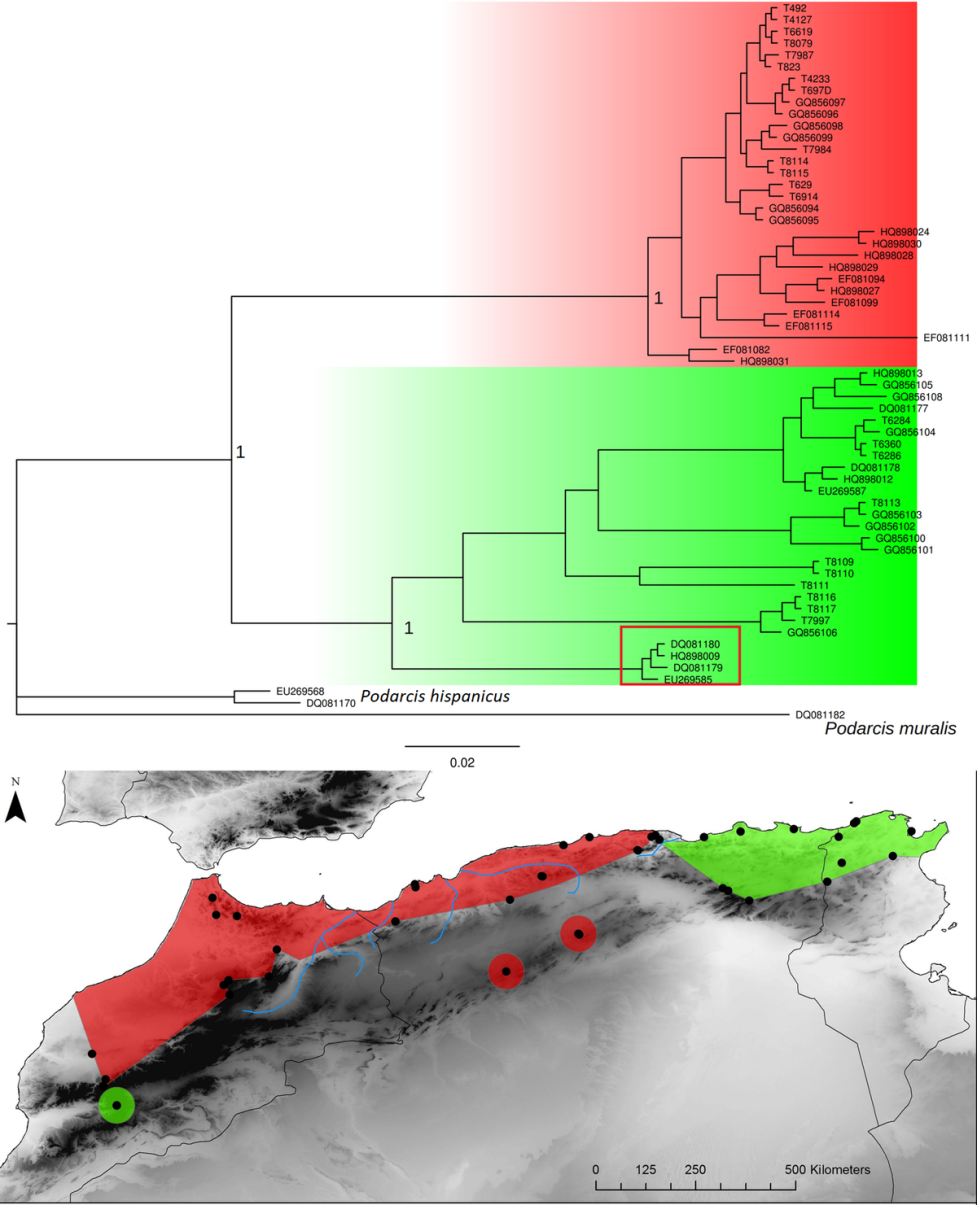
*Podarcis voucheri* Bayesian inference phylogenetic tree and geographic distribution of deep clades. The red rectangle in the phylogenetic tree highlights the Moroccan lineage falling in the eastern clade.

**Fig 8:**
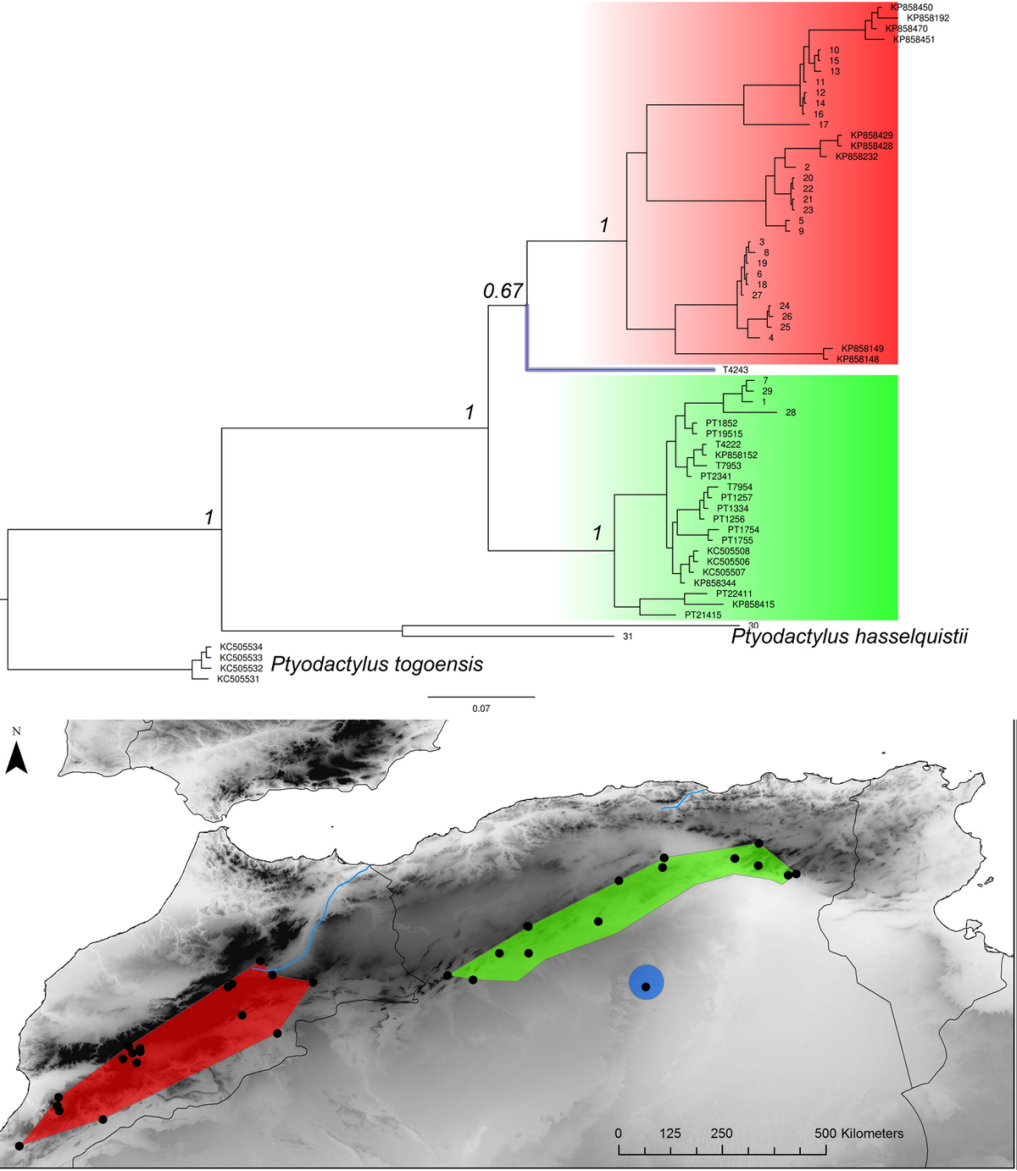
*Ptyodactylus oudrii* Bayesian inference phylogenetic tree and geographic distribution of deep clades. The phylogenetic position of the specimen from Ghardaia (the blue dot on the map and blue highlighted branch in the tree) is not supported.

**Fig 9:**
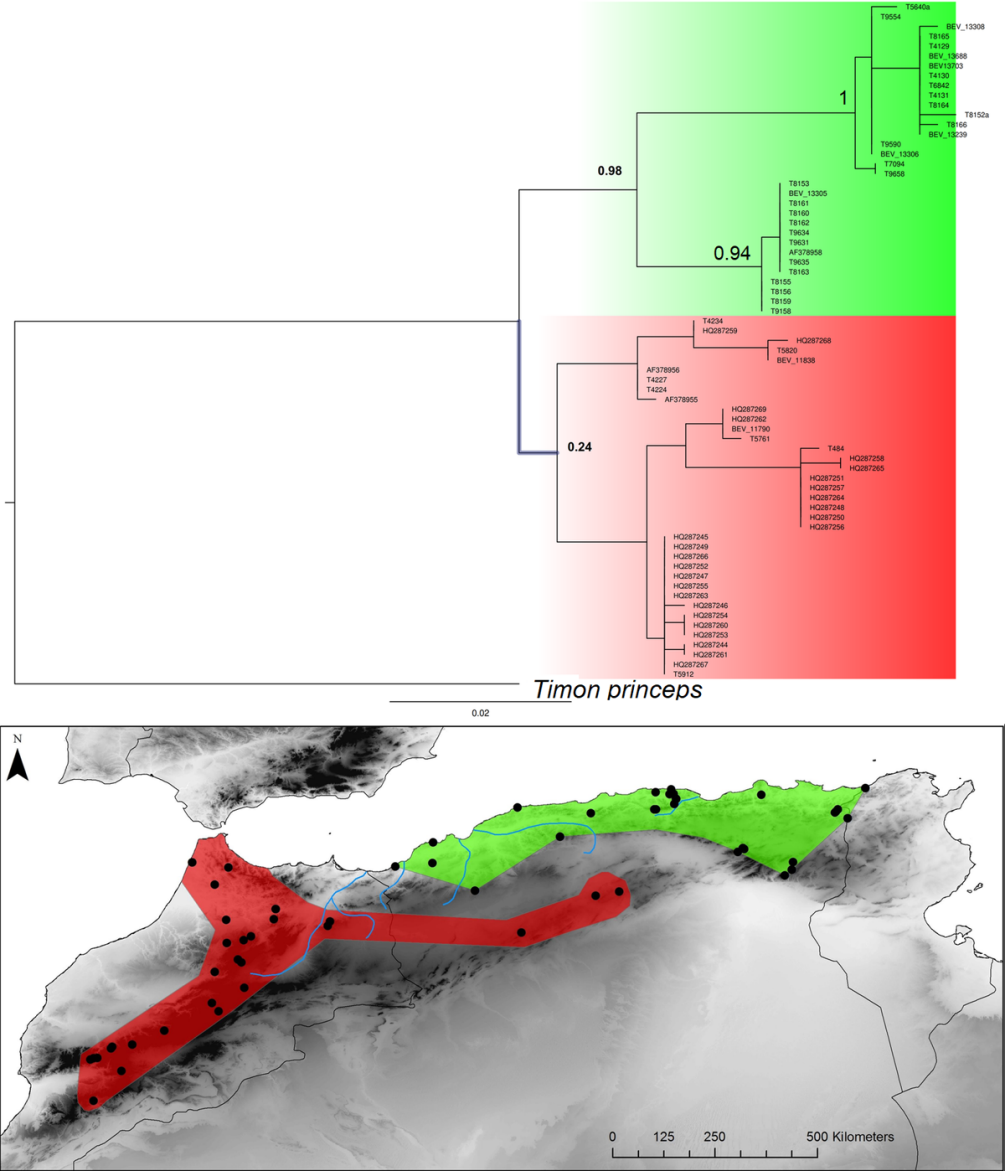
Bayesian inference phylogenetic tree and geographic distribution of deep clades. *Timon spp*. The reciprocal monophyly of E-W clades is not supported by any reconstruction method. However, the previous studies have shown that the Moroccan populations and the Tunisian ones are reciprocally monophyletic and highly supported (see references in the discussion).

**Fig 10:**
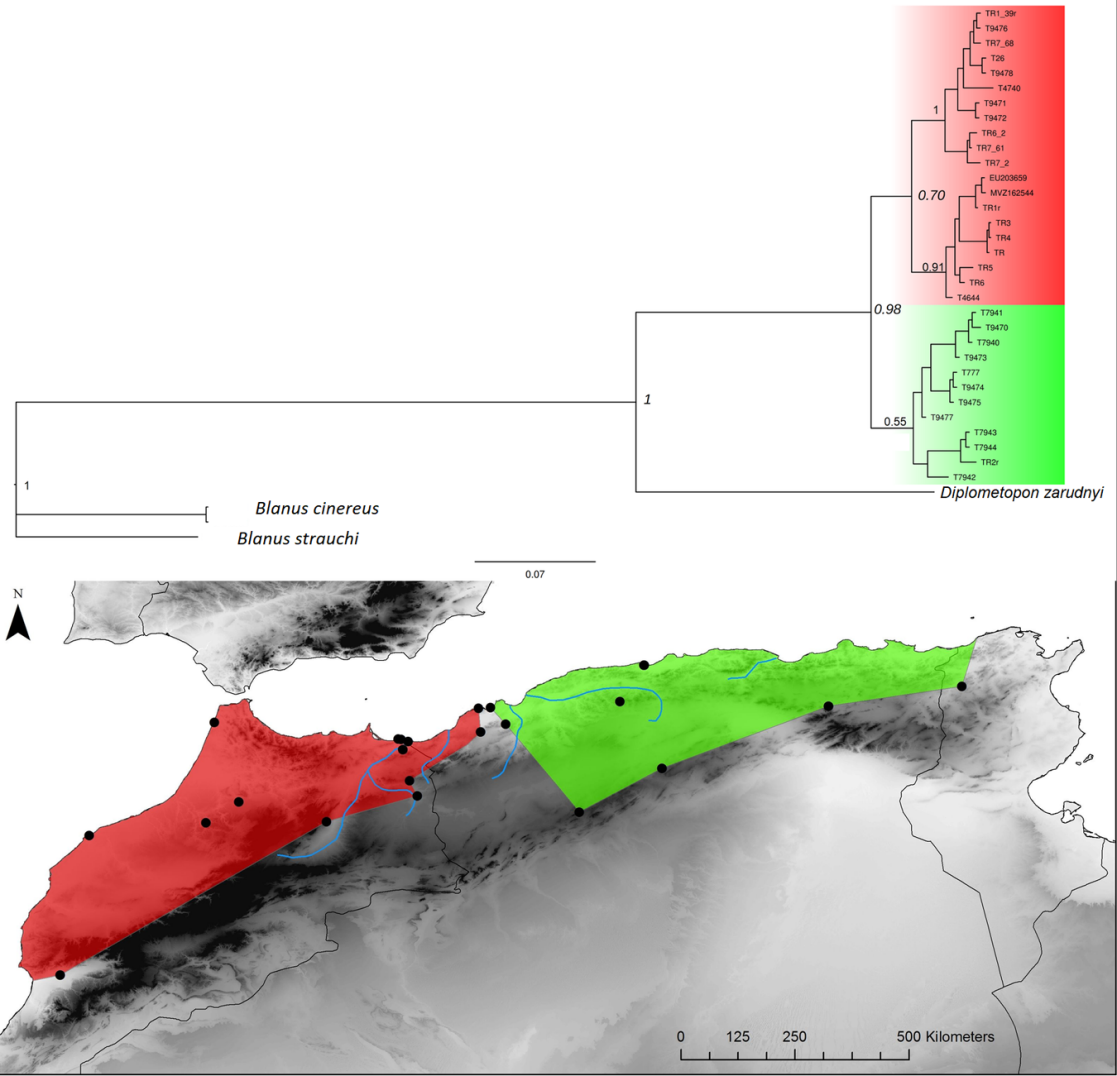
Trogonophis wiegmanni. Bayesian inference phylogenetic tree and geographic distribution of deep clades.

**Fig 11:**
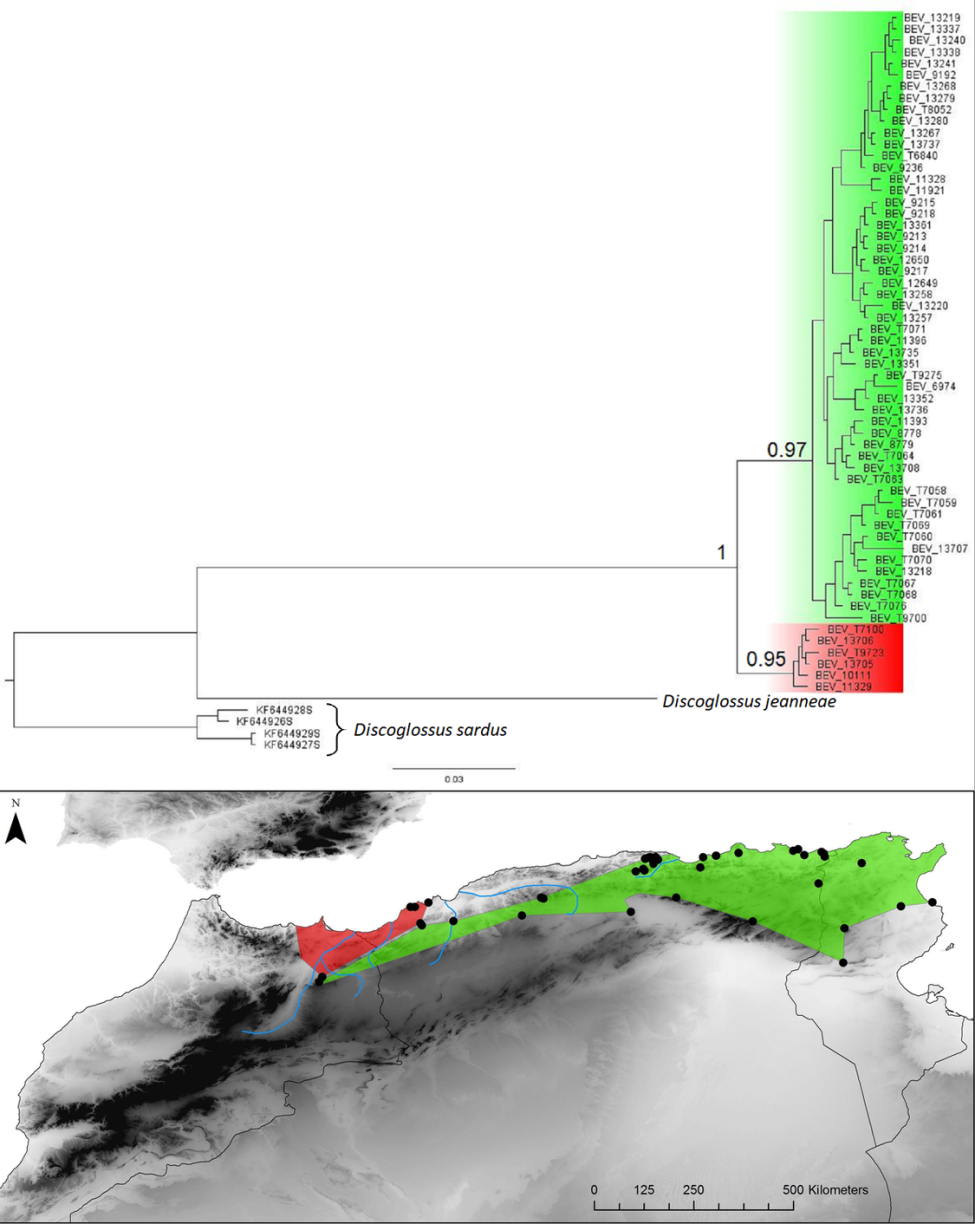
Discoglossus pictus. Bayesian inference phylogenetic tree and geographic distribution of deep clades.

**Fig 12:**
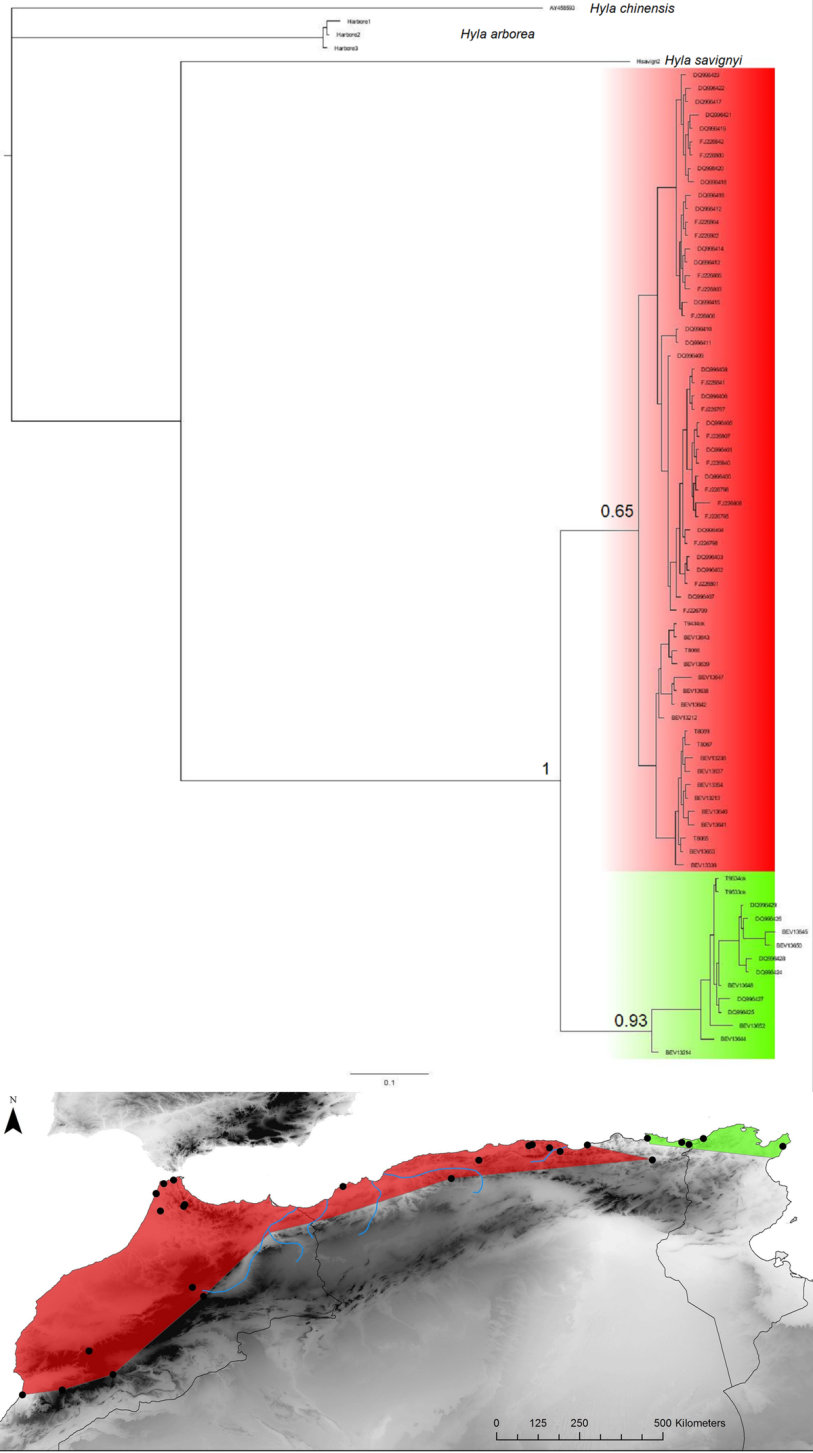
Hyla meridionalis. Bayesian inference phylogenetic tree and geographic distribution of deep clades.

**Fig 13:**
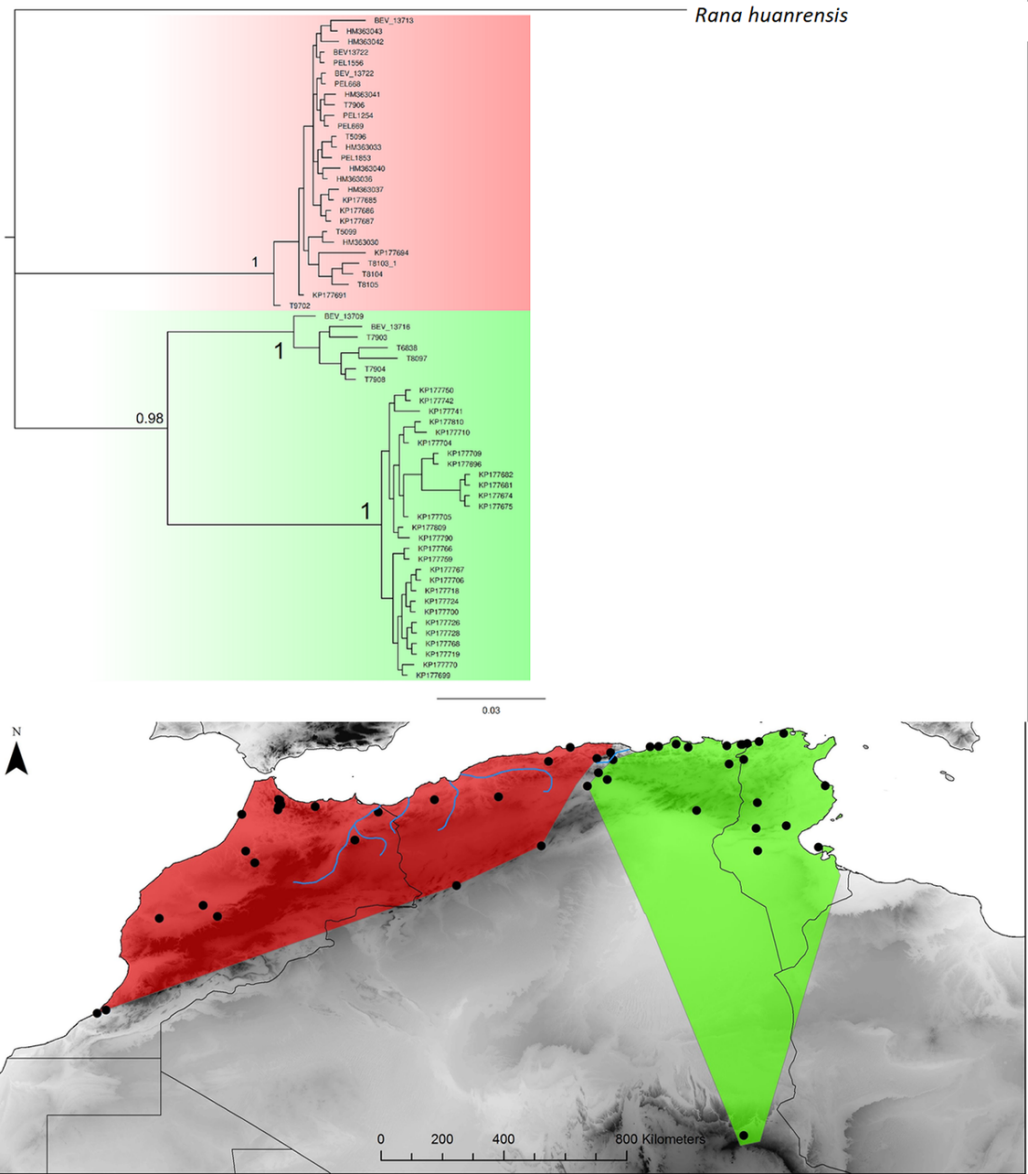
Pelophylax saharicus. Bayesian inference phylogenetic tree and geographic distribution of deep clades.

**Table 2:** Number of groups inferred by ABGD program, the sequences lengths, genetic distances between and within ABGD groups.

Taxon	ABGD	Length (pb)	Distance within group	Distance between groups
***Acanthodactylus erythrurus***	15	776	0–0.0156–0.0297	0.068–0.125–0.166
***Chalcides ocellatus***	2	425	0.0286–0.0338–0.039	0.102 –Nc– 0.102
***Hemorrhois hippocrepis***	2	641	0.0042–0.0046–0.0049	0.029 –Nc– 0.029
***Natrix maura***	5	604	0–0.0023–0.0052	0.025–0.045–0.057
***Podarcis vaucheri***	6	659	0.001–0.0129–0.0294	0.064–0.096–0.134
***Ptyodactylus oudrii***	9	454	0.0033–0.0107–0.0268	0.034–0.117–0.177
***Timon pater / tangitanus***	5	477	0.0009–0.0048–0.123	0.021–0.042–0.057
***Trogonophis wiegmanni***	3	474	0.0123–0.0148–0.01814	0.0123–0.0148–0.0446
***Discoglossus pictus***	2	964	0.007–0.008–0.009	0.021 –Nc– 0.021
***Hyla meridionalis***	4	775	0.0011–0.0049–0.0096	0.012–0.047–0.076
***Pelophylax saharicus***	3	654	0.0034–0.005–0.0068	0.072–0.108–0.13

From left to right the minimum, medium and maximum distances are given within and between groups.

In addition, a more complex situation emerges alongside the E-W general pattern in *Acanthodactylus* (an endemic lineage in the Atlas mountain of Morocco is of uncertain position and might be basal), *Podarcis* (a deeply divergent lineage from the Atlas Mountain of Morocco groups with a high support with the E clade, see REF) and *Ptyodactylus* (a basal lineage found in the pre-Sahara of Algeria is of uncertain position).

All studied taxa showed a high haplotypic diversity (Mean Hd = 0.094 ± 0.05, Table 3). The (E) and (W) clades don’t show a significant difference in Hd (Kruskal-Wallis chi-squared = 0.434, df = 1, p = 0.51, see Table 3).

**Table 3:** Relevant node ages and haplotypic diversity.

*Taxon*	Suture Zones	N	Gene	Lenght (pb)	S		H	Hd	Pi	MRCA (MY)		N	S	H	Hd	Pi
***Acanthodactylus*** ***erythrurus***	**M**	92	ND4 + tRNA	755	285		70	0.99	0.096	4.5 [3–6.25]	**Est**	32	208	23	0.97	0.08
											**West**	60	224	47	0.99	0.08
***Chalcides ocellatus***	**K**	51	CytB	425	113		44	0.99	0.06	4.57* [4.4–4.74]	**Est**	17	46	14	0.98	0.024
											**West**	34	98	30	0.98	0.036
***Hemorrhois*** ***hippocrepis***	**K**	35	Cytb	300	15		11	0.83	0.009	**_**	**Est**	9	5	3	0.55	0.007
											**West**	26	10	9	0.74	0.005
***Natrix maura***	**M**	48	ND4	604	49		17	0.87	0.025	9.2 [5.7–14]	**Est**	34	44	13	0.82	0.015
											**West**	14	6	5	0.59	0.0017
***Podarcis vaucheri***	**K**	54	ND4 + tRNA	659	180		39	0.98	0.075	2.2 [1.5–3]	**Est**	25	127	19	0.97	0.06
											**West**	29	69	20	0.95	0.018
***Ptyodactylus*** ***oudrii***	**M**	51	12S-RNA	350	92		28	0.96	0.093	10.6 [6.5–15.4]	**Est**	21	80	14	0.93	0.005
											**West**	30	82	18	0.95	0.008
***Timon spp***		69	16S-RNA	477	46		19	0.92	0.33	6.9 [3.8–11.4]	**Est**	32	27	8	0.8	0.024
											**West**	37	28	11	0.88	0.019
***Trogonophis*** ***wiegmanni***	**M**	30	16S-RNA	474	48		20	0.97	0.034	**_**	**Est**	12	19	7	0.89	0.017
											**West**	18	37	13	0.96	0.025
***Discoglossus*** ***pictus***	**M**	58	CytB	936	68		36	0.97	0.009	2 [1–3.3]	**Est**	52	52	31	0.96	0.006
											**West**	6	7	5	0.93	0.002
***Hyla*** ***meridionalis***	**E**	50	CO1	775	97		34	0.96	0.031	3 [1.5–5.76]	**Est**	10	22	9	0.97	0.009
											**West**	40	46	25	0.95	0.014
***Pelophylax*** ***saharicus***	**K**	63	CO1	654	114		33	0.93	0.066	3.65 [1.8–5.4]	**Est**	26	21	16	0.85	0.003
											**West**	37	64	17	0.87	0.028

N: Number of specimens for each taxon, gene: genetic marker, L: sequence length, S: number of segregating sites, H: number of haplotypes in the complete data set of each taxon, Hd: haplotypic diversity calculated by DnaSp, Pi: TMRCA: age of most recent ancestor of E-W clades.

*: calibrations obtained from [36]

### Location of E-W suture zones

**Fig 14:**
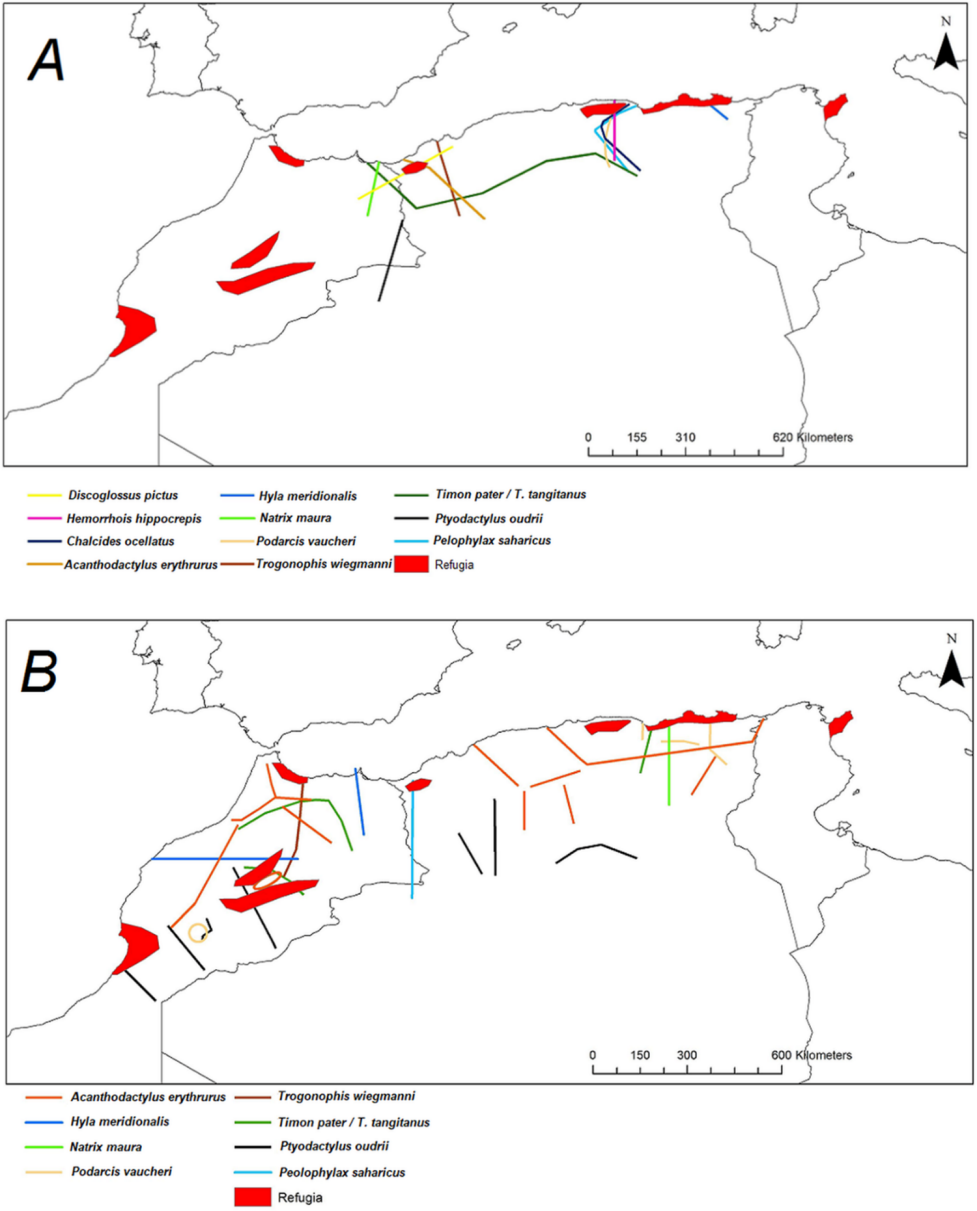
**A: Location of suture zones between deep E-W clades. B: Location of internal suture zones between ABGD groups.** A: Coloured lines correspond to the disjunction between E and W clades. B: Coloured lines correspond to the limits between ABGD lineages. Red areas correspond to biogeographical refugia described by [36].

Algerian populations belong to the E or W main lineages depending on the taxon (Fig 14). For most species the limits between the E and W lineages span a narrow longitudinal range but for *Timon*, specimens of the E lineages occupy the north of Algeria while populations of the W lineage are found along the Saharan Atlas in Algeria, resulting in a north-south phylogeographic divide within Algeria that contrast with the situation with the other taxa.

Two repeated patterns are identified for the location of the E-W phylogeographic breaks: the Moulouya suture zone around the Algeria-Morocco border and the Kabylian suture zone in the eastern part of Algeria (Fig 14). Six taxa out of 11 obey the “Moulouya pattern”, with suture zones located between 3.4 and 0.1°W around the Algerian-Moroccan boundaries; four species have suture zones in the Kabylia region between 3.6 and 5.3°E. *Hyla meridionalis* is the only species that deviates from these patterns as its disjunction area is located further east around 7.5°E. As can be seen from Fig 13, the limits in the Kabylian suture zone are more tightly spaced than around the Moulouya valley.

#### Divergence time of the E-W clades

Divergence times estimated for nine of the eleven taxa, as well as the 95% highest posterior densities (HPD), are reported in Table 3. Three main diversification periods can be identified (Table 3). The first period, represented by *P*. *oudrii*, *N*. *maura*, and *Timon*.*spp*., with an old diversification dated to the Miocene, long before the Messinian crisis. The second diversification event corresponds with the Messinian salinity crisis (*A*. *erythrurus* and *C*. *ocellatus*). The last period corresponds with more recent divergence within the Maghreb after the Messinian crisis between 2 and 3.6 MYA (*D*. *pictus*, *H*. *meridionalis*, *P*. *saharicus*, *P*. *vaucheri*)

### Geographical distribution of internal clades and secondary phylogeographic suture zones

As can be seen from Figs 1 and 2, the secondary suture zones between the internal clades delimited by ABGD do not follow a general trend as internal clade limits are spread over the whole of Algeria. The only common pattern is that most sutures are E-W breaks, reinforcing the idea that for Mediterranean or mesic species, longitudinal fragmentation of ancestral areas was the main force shaping divergence in mtDNA.

## Discussion

### How significant is the persistence of the E-W pattern after inclusion of Algerian samples?

The fact that we identified an E-W split as the most basal divergence within North Africa in most species (and that this E-W split structures most of the mitochondrial diversity in all of them) should not be interpreted as a support for the generality of the E-W divergence pattern in North Africa. We have chosen species where the E-W pattern had been previously recovered and we wanted to know if this pattern was disrupted by inclusion of Algerian samples. However, the persistence of this E-W divergence was not the only possible outcome of our study. In theory, new Algerian lineages could place at the basis of the mtDNA tree, leaving Moroccan and Tunisian samples in a monophyletic clade, if parts of Algeria harboured lineages that are older than the separation of Tunisian and Moroccan populations. The fact that we never recovered this pattern supports the idea that allopatry in E and W refugia is an important factor driving partitioning of genetic mitochondrial diversity in the Maghreb herpetofauna. A proper examination of this hypothesis would require phylogeographic studies of a random sample of species with a large Maghreban distribution, however.

A rapid survey of published phylogeography for Maghreban amphibians and reptiles uncovers one other general patterns in addition to the main E-W divergence: the deepest divergence is located in Morocco, with the rest of the Maghreb colonized by one of the Moroccan lineages (*Mauremys leprosa*: [76]; *Daboia mauritanica*: [77]; *Bufotes boulengeri*: [78]. The other taxa either follow the general E-W divergence pattern: *Testudo graeca*: [79], *Salamandra algira* [80–81], or their phylogeography within North Africa was too poorly resolved to assign them to one of these situations: *Sclerophrys mauritanica*: [82]; *Psammodromus algirus*: [83].

In addition, more complex patterns have also emerged aside the main E-W divergence in North Africa. For example, in *Timon spp*, the disjunction corresponds with the western break area in the northern part of the range but isolated populations along the Saharan atlas to east-central Algeria belong to the western clade. These populations are geographically separated from the northern ones in Algeria carrying the eastern mtDNA haplotypes by the arid areas of the High Plateaux. Isolated populations genetically assigned to the Eastern clade are sometimes found within the range of western populations, for example in *Podarcis*. Last, basal clades occur in the High Atlas Mountains (Morocco) for *A*. *erythrurus* or in the pre-Sahara for *P*. *oudrii*.

### Concordance of suture zones between E and W lineages in several taxa suggest common historical processes

The limits of the E and W clades are concentrated in two main areas: a suture zone around the boundaries of Algeria and Morocco, broadly corresponding with the previously suggested suture zone of the Moulouya valley and one newly identified disjunction zone in the Kabylia region (see [34,44]). Both areas are also suture zones between ABGD lineages for several species. This repeated pattern, supported by results of other studies, indicate that common historical processes shaped the current divergence around these two areas. Note that there is no apparent difference in TMRCA of E and W lineages between the two main disjunction zones (see Table 3).

The Kabylian break is located around the Soumam Valley in the centre-east of Algeria. It had not been identified before this study, even if *Podarcis vaucheri* [56] and *Cornu aspersum* [84] seemed to conform to this pattern. We show here that this area also constitutes a major divide for *Chalcides ocellatus*, *Hemorrhois hippocrepis* (Fig 1) and *Pelophylax saharicus* (Fig 2), hence for four out of 11 taxa. The Kabylian break is also the second main disjunction area for *Natrix maura* and *Timon pater* (Fig 1).

Six of our 11 taxa conform more or less strictly to the western break pattern (see Fig 1), while this area is also a secondary break for *Hyla meridonalis* and *Pelophylax saharicus* (Fig 2). This suture zone has been identified by several previous studies [34,44,70,85]. These studies suggested that this suture zone was a direct consequence of the Moulouya River valley and its arid corridor, acting as a barrier for Mediterranean species (but see below).

### Geographical barriers and glacial refugia

Our results suggest that the transition zone is wider than the Moulouya River valley itself (see Figs 1 and 2), suggesting that it cannot be the direct consequence of this valley. In addition, the W suture zone affects species with very diverse ecology, some of them occur in the Moulouya River valley itself (for ex. *Pelophylax*, *Natrix maura*, *Discoglossus*, *Acanthodactylus erythrurus*). It is thus obvious that the western suture zone cannot be explained by the Moulouya River itself acting as a barrier to dispersal. The Moulouya River is located in the Rifan corridor, a former marine corridor joining the Mediterranean east of the Rif Mountains with the Atlantic near present-day Rabat [86,87]. The width of this marine corridor was at least 60 km. The corridor opened between ~ 8.0 and 7.6 Ma, and the maximum sea level was reached in the late-Tortonian and decreased at the Tortonian-Messinian limit (7.2 MYA) but the connexion between the Mediterranean and the Atlantic continued during the Messinian. The final closure of the Rif corridor is dated to ~6.1 to 5.6 MYA with a large marine basin persisting until the end of the upper Pliocene 5,6 MYA [86,88–90].

The Rifan corridor was undoubtedly a major geographic barrier that split the Maghreb for a long period (Miocene to mid Pliocene) and several E-W divergences that we have identified can be dated to this period (see Table 3). The fact that the western break zone coincides with the area of the Rifan Corridor and that the molecular dating of these splits corresponds broadly to this period suggests that the distribution of many species were fragmented on either side of this corridor. This major past geographical barrier could explain East-west divergence in the North and North-South divergence for other species in the southern part of the corridor (Middle Atlas).

The Kabylian break is located around the Soumam valley. Although, like the Moulouya, the Soumam is a relatively dry valley compared to adjacent areas, several species that are currently affected by the Kabylian break inhabit the valley itself. We thus suggest that the Kabylian break is not a direct consequence of the presence of the Soumam River either. Unfortunately, the palaeogeography of the area is poorly documented, but no major barrier comparable to the Rifan Corridor has been identified in Kabylia.

As mentioned above (introduction), the role of the glacial / interglacial cycles in generating biodiversity below or around the species level has been thoroughly documented in Europe and North America. Nothing comparable exists for Northern Africa but we show here with a comparative phylogeography approach a good congruence between the Kabylia suture zones and glacial refugia identified by [20] on the basis of intra- specific phylogeographies of plant species.

The major paleo barrier of the Rifan Corridor could thus explain a fair proportion of ancient divergence events in Maghreban species but the origin of many cladogenesis events remain elusive. However, the conservation of these old divergent lineages requires a long term separation or a repeated expansion / contraction of the ranges of these lineages. Given the congruence in Kabylia between the location of suture zones and the location of plant refugia, the hypothesis of repeated contraction to glacial refugia seem the most likely scenario to explain the current diversity at species and lineage scales.

### Evolutionary significance of mitochondrial diversity

The lack of contemporary geographical barrier and the narrowness of the contact zones suggests the possibility of competitive exclusion between lineages. Such reciprocal exclusion can be neutral (drift in local demes) or selective (if the mtDNA lineages do not freely interbred and exclude each other). We need a multilocus approach to examine these hypotheses. Firstly, we need to verify that the mtDNA lineages that we have identified correspond with real evolutionary units, as profound mitochondrial divergence can sometime persist without nuclear divergence (see [91]). We already know that some of the mitochondrial divergence we have studied are mirrored in nuclear genes: for *Pelophylax*, the central and W lineages differ in allozymes [92], (no sample from the E lineage were included) and for *Podarcis* E and W lineages are highly divergent in allozymes [67]. Secondly, we need to assess whether these evolutionary units persist in the face of gene flow (implying restriction to dispersal) or are associated with speciation events. Indeed, several of the “species” that we have examined are probably made of several species, implying that current taxonomy is inadequate.

A better understanding of the role of speciation events in generating the pattern of diversity that we have exposed here would also have important consequences in conservation. If some of the lineages with restricted distribution that we identified in Algeria correspond to valid biological species, the level of endemism and conservation status of the Algerian herpetofauna would be significantly changed. Indeed, the western and eastern lineages of *Timon* are already widely recognized as two species (*T*. *tangitanus* in the west and *T*. *pater* in the east) even if this might require confirmation. A recent detailed investigation on *Tarentola mauritanica* in Morocco [93] identified several unrecognized species and *Ptyodactylus oudrii* has been suggested to constitute a species complex [39]. Many similar cases probably await discovery in Algeria, where the amount of detailed studies on small vertebrates is much lower and ABGD identified multiple lineages in several “species” that we investigated. While some of these lineages undoubtedly correspond with truly intraspecific diversity, a multilocus study is needed in their contact zones to figure out the level of reproductive isolation between these lineages.

### Impact of poorly resolved phylogenies

The mitochondrial phylogenies we used were sometimes poorly supported. While in several taxa, it was a consequence of us using short sequences and the original studies reported better supported phylogenies, in *Trogonophis*, the obtained phylogeny lacks strong nodal supports (posterior probability = 0.55), however a recent study resulted in the same overall topology with highly supported nodes [94]. In the case of *Timon spp*, the original study [42] reported a well-supported phylogeny but we were unable to recover the same topology with our own analyses of the same dataset whatever the method used. These cases highlight the need for improved phylogenetic resolution through longer mitochondrial sequences and addition of multilocus data.

## Supporting information

S1 Table The list of used tissue samples, their geographic origin and GenBank accession numbers. Latitudes (LAT) and Longitudes (LON) are given in WGS84 geodetic system. The locus is specified only when there is concatenation of 2 loci for the phylogenetic reconstruction, otherwise see the material and methods for the used marker.
(DOCX)

S2 Table List of primers, their references and hybridisation temperatures. (DOCX)
