# Supplementary material for: Comparative phylogeography of amphibians and reptiles in Algeria suggests common causes for the east-west phylogeographic breaks in the Maghreb
Source: PLoS One. 2018 Aug 29;13(8):e0201218. doi: 10.1371/journal.pone.0201218 (PMC6114291; doi:10.1371/journal.pone.0201218)
Supplement: S1 Table — Latitudes (LAT) and Longitudes (LON) are given in WGS84 geodetic system. The locus is specified only when there is concatenation of 2 loci for the phylogenetic reconstruction, otherwise see the material and methods for the used marker. (DOCX) [file pone.0201218.s001.docx]

**S1 Table. The list of used tissue samples, their geographic origin and GenBank accession numbers.** Latitudes (LAT) and Longitudes (LON) are given in WGS84 geodetic system. The locus is specified only when there is concatenation of 2 loci for the phylogenetic reconstruction, otherwise see the Materials and methods for the used marker.

| ***Species*** | **Code** | **Voucher** | **GeneBank accession** | **LAT** | **LON** | **Origin** |
| --- | --- | --- | --- | --- | --- | --- |
| *Chalcides ocellatus* | BEV.13254 | BEV.13254 | KY274205 | 36.712 | 5.548 | Present study |
| *Chalcides ocellatus* | BEV.13614 | BEV.13614 | KY274206 | 35.599 | 3.942 | Present study |
| *Chalcides ocellatus* | BEV.13613 | BEV.13613 | KY274207 | 35.052 | 4.135 | Present study |
| *Chalcides ocellatus* | T9580 | BEV.T9580 | KY274208 | 35.275 | -0.776 | Present study |
| *Chalcides ocellatus* | BEV.13611 | BEV.13611 | KY274209 | 35.275 | -0.776 | Present study |
| *Chalcides ocellatus* | T9548 | BEV.T9548 | KY274210 | 35.247 | 0.121 | Present study |
| *Chalcides ocellatus* | T8037 | BEV.T8037 | KY274211 | 35.444 | 1.602 | Present study |
| *Chalcides ocellatus* | T8045 | BEV.T8045 | KY274212 | 36.712 | 5.548 | Present study |
| *Chalcides ocellatus* | T8044 | BEV.T8044 | KY274213 | 36.57 | 4.538 | Present study |
| *Chalcides ocellatus* | T8043 | BEV.T8043 | KY274214 | 36.351 | 2.667 | Present study |
| *Chalcides ocellatus* | T8042 | BEV.T8042 | KY274215 | 36.315 | 2.722 | Present study |
| *Chalcides ocellatus* | T8041 | BEV.T8041 | KY274216 | 36.315 | 2.722 | Present study |
| *Chalcides ocellatus* | T8039 | BEV.T8039 | KY274217 | 35.05 | 1.482 | Present study |
| *Chalcides ocellatus* | T8038 | BEV.T8038 | KY274218 | 35.444 | 1.602 | Present study |
| *Chalcides ocellatus* | T8035 | BEV.T8035 | KY274219 | 35.05 | 1.482 | Present study |
| *Chalcides ocellatus* | T8034 | BEV.T8034 | KY274220 | 35.444 | 1.602 | Present study |
| *Chalcides ocellatus* | T8033 | BEV.T8033 | KY274221 | 35.778 | 6.076 | Present study |
| *Chalcides ocellatus* | T8032 | BEV.T8032 | KY274222 | 35.522 | 6.041 | Present study |
| *Chalcides ocellatus* | T8031 | BEV.T8031 | KY274223 | 35.946 | 6.52 | Present study |
| *Chalcides ocellatus* | T8030 | BEV.T8030 | KY274224 | 35.946 | 6.52 | Present study |
| *Chalcides ocellatus* | T8029 | BEV.T8029 | KY274225 | 36.014 | 6.57 | Present study |
| *Chalcides ocellatus* | T8028 | BEV.T8028 | KY274226 | 36.014 | 6.57 | Present study |
| *Chalcides ocellatus* | T8027 | BEV.T8027 | KY274227 | 36.785 | 5.608 | Present study |
| *Chalcides ocellatus* | T8026 | BEV.T8026 | KY274228 | 36.776 | 5.606 | Present study |
| *Chalcides ocellatus* | T7917 | BEV.T7917 | KY274229 | 34.55 | 2.798 | Present study |
| *Chalcides ocellatus* | T8064 | BEV.T8064 | KY274230 | 36.066 | 4.198 | Present study |
| *Chalcides ocellatus* | T9407 | BEV.T9407 | KY274231 | 35.654 | -0.977 | Present study |
| *Chalcides ocellatus* | Mordjadjo | Mordjadjo | KY274232 | 35.686 | -0.823 | Present study |
| *Chalcides ocellatus* | BEV.11048 | BEV.11048 | KY274233 | 33.567 | 1.172 | Present study |
| *Chalcides ocellatus* | T1608 | BEV.T1608 | KY274234 | 35.33 | -1.316 | Present study |
| *Chalcides ocellatus* | T1609 | BEV.T1609 | KY274235 | 36.936 | 6.886 | Present study |
| *Chalcides ocellatus* | T4125 | BEV.T4125 | KY274236 | 36.378 | 3.905 | Present study |
| *Chalcides ocellatus* | T4220 | BEV.T4220 | KY274237 | 33.565 | 1.171 | Present study |
| *Chalcides ocellatus* | T4225 | BEV.T4225 | KY274238 | 33.358 | 0.969 | Present study |
| *Chalcides ocellatus* | T4231 | BEV.T4231 | KY274239 | 34.418 | 3.48 | Present study |
| *Chalcides ocellatus* | T4242 | BEV.T4242 | KY274240 | 33.58 | 2.965 | Present study |
| *Chalcides ocellatus* | BEV.9200 | BEV.9200 | KY274241 | 36.747 | 3.005 | Present study |
| *Chalcides ocellatus* | BEV.9227 | BEV.9227 | KY274242 | 36.099 | 2.92 | Present study |
| *Chalcides ocellatus* | BEV.9203 | BEV.9203 | KY274243 | 36.594 | 2.572 | Present study |
| *Chalcides ocellatus* | T633 | BEV.T633 | KY274244 | 35.569 | -1.181 | Present study |
| *Chalcides ocellatus* | T635 | BEV.T635 | KY274245 | 35.575 | -1.145 | Present study |
| *Chalcides ocellatus* | T637 | BEV.T637 | KY274246 | 35.393 | -0.3 | Present study |
| *Chalcides ocellatus* | BEV.9255 | BEV.9255 | KY274247 | 35.669 | -0.4 | Present study |
| *Chalcides ocellatus* | T6500 | BEV.T6500 | KY274248 | 36.572 | 4.533 | Present study |
| *Chalcides ocellatus* | T6839 | BEV.T6839 | KY274249 | 36.626 | 4.5 | Present study |
| *Chalcides ocellatus* | T4216 | BEV.T4216 | KY274250 | 35.827 | -0.483 | Present study |
| *Chalcides ocellatus* | BEV.13612 | BEV.13612 | KY274251 | 31.936 | 2.456 | Present study |
| *Hemorrhois hippocrepis* | BEV.T1610 | BEV.T1610 | KY513292 | 35.661 | -0.849 | Present study |
| *Hemorrhois nummifer* | BEV.T7863 | BEV.T7863 | KY513297 | 32.461 | 50.164 | Present study |
| *Hemorrhois algirus* | BEV.13250 | BEV.13250 | KY513298 | 34.109 | 2.999 | Present study |
| *Hemorrhois algirus* | BEV.T9629 | BEV.T9629 | KY513308 | 34.939 | 6.931 | Present study |
| *Hemorrhois hippocrepis* | BEV.T619 | BEV.T619 | KY513293 | 35.661 | -0.849 | Present study |
| *Hemorrhois hippocrepis* | BEV.9253 | BEV.9253 | KY513294 | 35.801 | -0.258 | Present study |
| *Hemorrhois hippocrepis* | BEV.9378 | BEV.9378 | KY513295 | 20.557 | -12.63 | Present study |
| *Hemorrhois hippocrepis* | BEV.T7097 | BEV.T7097 | KY513296 | 35.548 | -1.169 | Present study |
| *Hemorrhois hippocrepis* | BEV.T8057 | BEV.T8057 | KY513299 | 35.34 | 1.236 | Present study |
| *Hemorrhois hippocrepis* | BEV.T8058 | BEV.T8058 | KY513300 | 35.537 | 2.913 | Present study |
| *Hemorrhois hippocrepis* | BEV.T8059 | BEV.T8059 | KY513301 | 36.802 | 5.651 | Present study |
| *Hemorrhois hippocrepis* | BEV.T8060 | BEV.T8060 | KY513302 | 36.184 | 6.585 | Present study |
| *Hemorrhois hippocrepis* | BEV.T8062 | BEV.T8062 | KY513303 | 36.696 | 4.339 | Present study |
| *Hemorrhois hippocrepis* | BEV.T8063 | BEV.T8063 | KY513304 | 36.758 | 4.509 | Present study |
| *Hemorrhois hippocrepis* | BEV.T8081 | BEV.T8081 | KY513305 | 35.381 | -1.235 | Present study |
| *Hemorrhois hippocrepis* | BEV.T8119 | BEV.T8119 | KY513306 | 35.634 | -0.776 | Present study |
| *Hemorrhois hippocrepis* | BEV.13696 | BEV.13696 | KY513307 | 32.768 | 0.553 | Present study |
| *Hemorrhois hippocrepis* | BEV.T9661 | BEV.T9661 | KY513309 | 35.804 | 1.625 | Present study |
| *Hemorrhois hippocrepis* | BEV.13695 | BEV.13695 | KY513310 | 33.793 | 0.747 | Present study |
| *Hemorrhois hippocrepis* | BEV.T8061 | BEV.T8061 | KY513311 | 36.777 | 8.374 | Present study |
| *Hyla meridionalis* | T8069 | BEV.T8069 | KY274253 | 36.366 | 2.692 | Present study |
| *Hyla meridionalis* | BEV13354 | BEV.13354 | KY274254 | 36.785 | 4.14 | Present study |
| *Hyla meridionalis* | BEV13238 | BEV.13238 | KY274255 | 36.697 | 4.603 | Present study |
| *Hyla meridionalis* | BEV13213 | BEV.13213 | KY274256 | 36.785 | 36.785 | Present study |
| *Hyla meridionalis* | BEV13212 | BEV.13212 | KY274257 | 36.774 | 5.629 | Present study |
| *Hyla meridionalis* | BEV13339 | BEV.13339 | KY274258 | 35.87 | 1.947 | Present study |
| *Hyla meridionalis* | T8065 | BEV.T8065 | KY274259 | 36.366 | 2.692 | Present study |
| *Hyla meridionalis* | T8066 | BEV.T8066 | KY274260 | 36.366 | 2.692 | Present study |
| *Hyla meridionalis* | T8067 | BEV.T8067 | KY274261 | 36.366 | 2.692 | Present study |
| *Hyla meridionalis* | BEV13644 | BEV.13644 | KY274262 | 36.955 | 7.252 | Present study |
| *Hyla meridionalis* | BEV13645 | BEV.13645 | KY274263 | 36.955 | 7.252 | Present study |
| *Hyla meridionalis* | BEV13650 | BEV.13650 | KY274264 | 36.789 | 8.377 | Present study |
| *Hyla meridionalis* | BEV13652 | BEV.13652 | KY274265 | 36.789 | 8.377 | Present study |
| *Hyla meridionalis* | BEV13653 | BEV.13653 | KY274266 | 36.793 | 8.374 | Present study |
| *Hyla meridionalis* | BEV13639 | BEV.13639 | KY274267 | 36.757 | 4.045 | Present study |
| *Hyla meridionalis* | BEV13640 | BEV.13640 | KY274268 | 36.757 | 4.045 | Present study |
| *Hyla meridionalis* | BEV13641 | BEV.13641 | KY274269 | 36.757 | 4.045 | Present study |
| *Hyla meridionalis* | BEV13637 | BEV.13637 | KY274270 | 35.654 | -0.977 | Present study |
| *Hyla meridionalis* | BEV13638 | BEV.13638 | KY274271 | 35.654 | -0.977 | Present study |
| *Hyla meridionalis* | BEV13643 | BEV.13643 | KY274272 | 36.601 | 4.893 | Present study |
| *Hyla meridionalis* | BEV13648 | BEV.13648 | KY274273 | 36.847 | 8.179 | Present study |
| *Hyla meridionalis* | T9534 | BEV.T9534 | KY274274 | 36.555 | 7.531 | Present study |
| *Hyla meridionalis* | T9533 | BEV.T9533 | KY274275 | 36.555 | 7.531 | Present study |
| *Hyla meridionalis* | BEV13642 | BEV.13642 | KY274276 | 36.601 | 4.893 | Present study |
| *Hyla meridionalis* | BEV13647 | BEV.13647 | KY274252 | 36.375 | 7.385 | Present study |
| *Ptyodactylus oudrii* | T7953 | BEV.13300 | KY576555 | 33.866 | 2.739 | Present study |
| *Ptyodactylus oudrii* | T7954 | BEV.13301 | KY576554 | 33.866 | 2.739 | Present study |
| *Ptyodactylus oudrii* | PT21415 | BEV.13666 | KY576565 | 33.177 | 0.587 | Present study |
| *Ptyodactylus oudrii* | PT2341 | BEV.13668 | KY576567 | 33.175 | 1.225 | Present study |
| *Ptyodactylus oudrii* | PT1256 | BEV.13669 | KY576558 | 35.246 | 4.167 | Present study |
| *Ptyodactylus oudrii* | PT1257 | BEV.13670 | KY576559 | 35.246 | 4.167 | Present study |
| *Ptyodactylus oudrii* | PT1334 | BEV.13671 | KY576560 | 35.038 | 4.131 | Present study |
| *Ptyodactylus oudrii* | PT1754 | BEV.13673 | KY576561 | 35.08 | 6.208 | Present study |
| *Ptyodactylus oudrii* | PT1755 | BEV.13674 | KY576562 | 35.08 | 6.208 | Present study |
| *Ptyodactylus oudrii* | PT1852 | BEV.13676 | KY576563 | 34.897 | 7.031 | Present study |
| *Ptyodactylus oudrii* | PT19515 | BEV.13677 | KY576564 | 34.874 | 6.857 | Present study |
| *Ptyodactylus oudrii* | T4222 | BEV.T4222 | KY576557 | 33.761 | 1.202 | Present study |
| *Ptyodactylus oudrii* | T4243 | BEV.T4243 | KY576556 | 32.443 | 3.769 | Present study |
| *Ptyodactylus oudrii* | PT22411 | BEV.T9562 | KY576566 | 32.601 | 0.015 | Present study |
| *Timon pater* | BEV.11790 | BEV.11790 | KY576568 | 31.283 | -7.383 | Present study |
| *Timon tangitanus* | BEV.11838 | BEV.11838 | KY576569 | 35.285 | -6.062 | Present study |
| *Timon pater* | BEV.13239 | BEV.13239 | KY576570 | 36.782 | 4.426 | Present study |
| *Timon pater* | BEV.13305 | BEV.13305 | KY576571 | 35.599 | 6.037 | Present study |
| *Timon pater* | BEV.13306 | BEV.13306 | KY576572 | 35.846 | 2.018 | Present study |
| *Timon pater* | BEV.13308 | BEV.13308 | KY576573 | 36.79 | 4.515 | Present study |
| *Timon pater* | BEV.13688 | BEV.13688 | KY576574 | 36.826 | 4.114 | Present study |
| *Timon pater* | BEV.13703 | BEV.13703 | KY576575 | 36.886 | 4.456 | Present study |
| *Timon pater* | T4129 | BEV.T4129 | KY576576 | 36.447 | 4.124 | Present study |
| *Timon pater* | T4130 | BEV.T4130 | KY576577 | 36.447 | 4.124 | Present study |
| *Timon pater* | T4131 | BEV.T4131 | KY576578 | 36.447 | 4.124 | Present study |
| *Timon pater* | T4224 | BEV.T4224 | KY576579 | 33.743 | 1.169 | Present study |
| *Timon pater* | T4227 | BEV.T4227 | KY576580 | 34.641 | 3.318 | Present study |
| *Timon tangitanus* | T4234 | BEV.T4234 | KY576581 | 34.552 | 2.796 | Present study |
| *Timon pater* | T484 | BEV.T484 | KY576582 | 31.236 | -7.82 | Present study |
| *Timon pater* | T5761 | BEV.T5761 | KY576583 | 31.283 | -7.383 | Present study |
| *Timon tangitanus* | T5820 | BEV.T5820 | KY576584 | 35.285 | -6.062 | Present study |
| *Timon pater* | T5912 | BEV.T5912 | KY576585 | 33.573 | -4.932 | Present study |
| *Timon pater* | T6842 | BEV.T6842 | KY576586 | 36.451 | 4.089 | Present study |
| *Timon pater* | T7094 | BEV.T7094 | KY576587 | 35.27 | -0.784 | Present study |
| *Timon pater* | T8152 | BEV.T8152 | KY576588 | 36.568 | 4.531 | Present study |
| *Timon pater* | T8153 | BEV.T8153 | KY576589 | 36.773 | 6.438 | Present study |
| *Timon pater* | T8155 | BEV.T8155 | KY576590 | 35.58 | 6.065 | Present study |
| *Timon pater* | T8156 | BEV.T8156 | KY576591 | 35.516 | 5.923 | Present study |
| *Timon pater* | T8158 | BEV.T8158 | KY576600 | 35.599 | 6.037 | Present study |
| *Timon pater* | T8159 | BEV.T8159 | KY576592 | 35.599 | 6.037 | Present study |
| *Timon pater* | T8160 | BEV.T8160 | KY576593 | 36.451 | 8.121 | Present study |
| *Timon pater* | T8161 | BEV.T8161 | KY576594 | 36.403 | 8.076 | Present study |
| *Timon pater* | T8162 | BEV.T8162 | KY576595 | 36.381 | 8.059 | Present study |
| *Timon pater* | T8163 | BEV.T8163 | KY576596 | 36.252 | 8.339 | Present study |
| *Timon pater* | T8164 | BEV.T8164 | KY576597 | 36.683 | 4.56 | Present study |
| *Timon pater* | T8165 | BEV.T8165 | KY576598 | 36.681 | 4.573 | Present study |
| *Timon pater* | T8166 | BEV.T8166 | KY576599 | 36.366 | 2.693 | Present study |
| *Timon pater* | T9554 | BEV.T9554 | KY576601 | 34.665 | 0.149 | Present study |
| *Timon pater* | T9590 | BEV.T9590 | KY576602 | 36.491 | 1.08 | Present study |
| *Timon pater* | T9631 | BEV.T9631 | KY576603 | 34.998 | 6.955 | Present study |
| *Timon pater* | T9634 | BEV.T9634 | KY576604 | 35.123 | 7.107 | Present study |
| *Timon pater* | T9635 | BEV.T9635 | KY576605 | 35.291 | 7.136 | Present study |
| *Timon pater* | T9658 | BEV.T9658 | KY576606 | 35.196 | -1.602 | Present study |
| *Trogonophis wiegmanni* | T4644 | BEV.11211 | KY576623 | 33.477 | -6,1988 | Present study |
| *Trogonophis wiegmanni* | T4740 | BEV.11304 | KY576620 | 35.134 | -2.401 | Present study |
| *Trogonophis wiegmanni* | T7940 | BEV.13288 | KY576610 | 34.551 | 2.797 | Present study |
| *Trogonophis wiegmanni* | T7941 | BEV.13289 | KY576611 | 34.551 | 2.797 | Present study |
| *Trogonophis wiegmanni* | T7942 | BEV.13290 | KY576617 | 35.866 | 1.968 | Present study |
| *Trogonophis wiegmanni* | T7943 | BEV.13291 | KY576615 | 35.778 | 6.076 | Present study |
| *Trogonophis wiegmanni* | T7944 | BEV.13292 | KY576616 | 35.778 | 6.076 | Present study |
| *Trogonophis wiegmanni* | T9470 | BEV.13678 | KY576612 | 33.688 | 1.174 | Present study |
| *Trogonophis wiegmanni* | T9471 | BEV.13679 | KY576621 | 35.275 | -0.776 | Present study |
| *Trogonophis wiegmanni* | T9472 | BEV.13680 | KY576622 | 35.275 | -0.776 | Present study |
| *Trogonophis wiegmanni* | T9473 | BEV.13681 | KY576613 | 35.748 | -0.575 | Present study |
| *Trogonophis wiegmanni* | T9474 | BEV.13682 | KY576609 | 36.595 | 2.441 | Present study |
| *Trogonophis wiegmanni* | T9475 | BEV.13683 | KY576608 | 36.595 | 2.441 | Present study |
| *Trogonophis wiegmanni* | T9476 | BEV.13684 | KY576618 | 35.744 | -0.822 | Present study |
| *Trogonophis wiegmanni* | T9477 | BEV.13685 | KY576614 | 35.416 | -0.282 | Present study |
| *Trogonophis wiegmanni* | T9478 | BEV.13686 | KY576619 | 35.084 | -2.209 | Present study |
| *Trogonophis wiegmanni* | T777 | BEV.9202 | KY576607 | 36.592 | 2.446 | Present study |
| *Natrix maura* | T9710 | BEV.T9710 | KY576624 | 36.87 | 7.425 | Present study |
| *Natrix maura* | T9705 | BEV.T9705 | KY576625 | 36.811 | 8.337 | Present study |
| *Natrix maura* | T9703 | BEV.T9703 | KY576626 | 36.827 | 7.922 | Present study |
| *Natrix maura* | T9701 | BEV.T9701 | KY576627 | 36.792 | 7.919 | Present study |
| *Natrix maura* | T9699 | BEV.T9699 | KY576628 | 36.789 | 8.377 | Present study |
| *Natrix maura* | T9663 | BEV.T9663 | KY576629 | 35.865 | 1.684 | Present study |
| *Natrix maura* | T9646 | BEV.T9646 | KY576630 | 36.823 | 3.663 | Present study |
| *Natrix maura* | T9591 | BEV.T9591 | KY576631 | 36.144 | 0.301 | Present study |
| *Natrix maura* | T9540 | BEV.T9540 | KY576632 | 35.638 | -1.06 | Present study |
| *Natrix maura* | T9486 | BEV.13694 | KY576633 | 36.782 | 8.116 | Present study |
| *Natrix maura* | T9484 | BEV.13692 | KY576635 | 34.551 | 2.796 | Present study |
| *Natrix maura* | T9483 | BEV.13691 | KY576636 | 35.27 | -0.772 | Present study |
| *Natrix maura* | T9482 | BEV.13690 | KY576637 | 35.239 | -0.733 | Present study |
| *Natrix maura* | T8084 | BEV.T8084 | KY576638 | 35.865 | 1.968 | Present study |
| *Natrix maura* | T8083 | BEV.T8083 | KY576639 | 36.403 | 8.076 | Present study |
| *Natrix maura* | T8082 | BEV.T8082 | KY576640 | 36.84 | 8.438 | Present study |
| *Natrix maura* | T7099 | BEV.T7099 | KY576641 | 35.491 | -0.221 | Present study |
| *Natrix maura* | T5543 | BEV.11555 | KY576642 | 34.159 | -6.68 | Present study |
| *Natrix maura* | T494 | BEV.T494 | KY576643 | 36.794 | 5.668 | Present study |
| *Natrix maura* | T4777 | BEV.11339 | KY576644 | 34.009 | -3.026 | Present study |
| *Natrix maura* | T4761 | BEV.T4761 | KY576645 | 34.358 | -2.047 | Present study |
| *Natrix maura* | T4612 | BEV.T4612 | KY576646 | 31.589 | -9.206 | Present study |
| *Natrix maura* | T6843 | BEV.T6843 | KY576648 | 36.629 | 4.505 | Present study |
| *Natrix maura* | T9485 | BEV.13693 | KY576634 | 36.782 | 8.116 | Present study |
| *Natrix maura* | T4128 | BEV.T4128 | KY576647 | 36.459 | 4.109 | Present study |
| *Podarcis vaucheri* | T4127 | BEV.T4127 | KY576662 | 4.123 | 36.447 | Present study |
| *Podarcis vaucheri* | T4233 | BEV.T4233 | KY576649 | 2.796 | 34.552 | Present study |
| *Podarcis vaucheri* | T492 | BEV.T492 | KY576650 | 2.446 | 36.567 | Present study |
| *Podarcis vaucheri* | T6284 | BEV.12373 | KY576651 | 8.992 | 37.064 | Present study |
| *Podarcis vaucheri* | T6286 | BEV.T6286 | KY576667 | 8.992 | 37.064 | Present study |
| *Podarcis vaucheri* | T629 | BEV.T629 | KY576663 | -0.906 | 35.687 | Present study |
| *Podarcis vaucheri* | T6360 | BEV.12374 | KY576652 | 8.992 | 37.064 | Present study |
| *Podarcis vaucheri* | T6619 | BEV.12654 | KY576664 | 4.11 | 36.458 | Present study |
| *Podarcis vaucheri* | T6914 | BEV.12666 | KY576668 | -0.889 | 35.622 | Present study |
| *Podarcis vaucheri* | T697 | BEV.9384 | KY576665 | 2.781 | 34.569 | Present study |
| *Podarcis vaucheri* | T7984 | BEV.13332 | KY576653 | 1.238 | 35.341 | Present study |
| *Podarcis vaucheri* | T7987 | BEV.13335 | KY576654 | 4.515 | 36.79 | Present study |
| *Podarcis vaucheri* | T7997 | BEV.13346 | KY576655 | 4.608 | 36.693 | Present study |
| *Podarcis vaucheri* | T8079 | BEV.T8079 | KY576669 | 3.028 | 36.749 | Present study |
| *Podarcis vaucheri* | T8109 | BEV.T8109 | KY576656 | 5.608 | 36.745 | Present study |
| *Podarcis vaucheri* | T8110 | BEV.T8110 | KY576657 | 5.608 | 36.745 | Present study |
| *Podarcis vaucheri* | T8111 | BEV.T8111 | KY576658 | 6.433 | 36.875 | Present study |
| *Podarcis vaucheri* | T8113 | BEV.T8113 | KY576659 | 6.037 | 35.599 | Present study |
| *Podarcis vaucheri* | T8114 | BEV.T8114 | KY576666 | 1.968 | 35.865 | Present study |
| *Podarcis vaucheri* | T8115 | BEV.T8115 | KY576670 | 1.947 | 35.872 | Present study |
| *Podarcis vaucheri* | T8116 | BEV.T8116 | KY576660 | 4.608 | 36.693 | Present study |
| *Podarcis vaucheri* | T8117 | BEV.T8117 | KY576661 | 4.608 | 36.693 | Present study |
| *Podarcis vaucheri* | T823 | BEV.9198 | KY576671 | 2.446 | 36.567 | Present study |
| *Acanthodactylus erythrurus belli* | T7963 | BEV.13311 | KY490405 | 35.764 | 2.131 | Present study |
| *Acanthodactylus erythrurus belli* | BEV.13560 | BEV.13560 | KY490386 | 35.644 | -0.986 | Present study |
| *Acanthodactylus erythrurus belli* |  | BEV.13561 | KY490389 | 34.427 | -0.939 | Present study |
| *Acanthodactylus erythrurus belli* | BEV.13562 | BEV.13562 | KY490390 | 34.427 | -0.939 | Present study |
| *Acanthodactylus erythrurus belli* | BEV.13563 | BEV.13563 | KY490394 | 36.699 | 2.805 | Present study |
| *Acanthodactylus erythrurus belli* | BEV.13564 | BEV.13564 | KY490388 | 35.274 | 6.486 | Present study |
| *Acanthodactylus sp* | BEV.13566 | BEV.13566 | KY490391 | 34.304 | 1.945 | Present study |
| *Acanthodactylus sp* | BEV.13567 | BEV.13567 | KY490393 | 34.304 | 1.945 | Present study |
| *Acanthodactylus erythrurus belli* | BEV.13570 | BEV.13570 | KY490398 | 36.043 | 4.168 | Present study |
| *Acanthodactylus erythrurus belli* | BEV.13571 | BEV.13571 | KY490399 | 36.914 | 8.343 | Present study |
| *Acanthodactylus erythrurus belli* | BEV.13572 | BEV.13572 | KY490395 | 36.846 | 7.937 | Present study |
| *Acanthodactylus erythrurus belli* | BEV.13573 | BEV.13573 | KY490397 | 36.935 | 7.193 | Present study |
| *Acanthodactylus erythrurus belli* | BEV.13574 | BEV.13574 | KY490396 | 36.935 | 7.193 | Present study |
| *Acanthodactylus erythrurus belli* | A2543 | BEV.13575 | KY490385 | 36.935 | 7.193 | Present study |
| *Acanthodactylus blanci* | BEV.13576 | BEV.13576 | KY490384 | 34.998 | 6.955 | Present study |
| *Acanthodactylus blanci* | BEV.13578 | BEV.13578 | KY490383 | 34.985 | 6.939 | Present study |
| *Acanthodactylus sp* | BEV.13587 | BEV.13587 | KY490392 | 34.304 | 1.945 | Present study |
| *Acanthodactylus erythrurus belli* | T6620 | BEV.T6620 | KY490400 | 36.808 | 3.592 | Present study |
| *Acanthodactylus erythrurus belli* | T6621 | BEV.T6621 | KY490401 | 36.885 | 4.523 | Present study |
| *Acanthodactylus erythrurus belli* | T6622 | BEV.T6622 | KY490402 | 36.885 | 4.522 | Present study |
| *Acanthodactylus erythrurus belli* | T6623 | BEV.T6623 | KY490403 | 36.885 | 4.523 | Present study |
| *Acanthodactylus erythrurus belli* | T6624 | BEV.T6624 | KY490404 | 36.885 | 4.522 | Present study |
| *Acanthodactylus erythrurus belli* | T7964 | BEV.T7964 | KY490406 | 36.868 | 6.077 | Present study |
| *Acanthodactylus erythrurus belli* | T7965 | BEV.T7965 | KY490407 | 36.868 | 6.077 | Present study |
| *Acanthodactylus erythrurus belli* | T7966 | BEV.T7966 | KY490408 | 36.815 | 4.973 | Present study |
| *Acanthodactylus erythrurus belli* | T7967 | BEV.T7967 | KY490409 | 36.624 | 4.852 | Present study |
| *Acanthodactylus erythrurus belli* | T8008 | BEV.T8008 | KY490410 | 34.597 | 3.102 | Present study |
| *Acanthodactylus erythrurus belli* | BEV.T9546 | BEV.T9546 | KY490387 | 34.665 | 0.149 | Present study |
| *Pelophylax saharicus* | T9501 | BEV.13709 | KY490358 | 32.731 | -0.004 | Present study |
| *Pelophylax saharicus* | BEV.13711 | BEV.13711 | KY490380 | 35.244 | -0.653 | Present study |
| *Pelophylax saharicus* | pel1254 | BEV.13712 | KY490374 | 35.832 | 4.416 | Present study |
| *Pelophylax saharicus* | T9505 | BEV.13713 | KY490359 | 35.832 | 4.416 | Present study |
| *Pelophylax saharicus* | T9506 | BEV.13714 | KY490375 | 35.65 | 3.834 | Present study |
| *Pelophylax saharicus* | T9508 | BEV.13716 | KY490360 | 36.777 | 3.335 | Present study |
| *Pelophylax saharicus* | BEV.13720 | BEV.13720 | KY490381 | 35.329 | 1.23 | Present study |
| *Pelophylax saharicus* | BEV.13721 | BEV.13721 | KY490379 | 35.329 | 1.23 | Present study |
| *Pelophylax saharicus* | T9514 | BEV.13722 | KY490362 | 36.776 | 6.788 | Present study |
| *Pelophylax saharicus* | T9514 | BEV.13722 | KY490362 |  |  | Present study |
| *Pelophylax saharicus* | T5096 | BEV.T5096 | KY490363 | 37.185 | 9.581 | Present study |
| *Pelophylax saharicus* | T5099 | BEV.T5099 | KY490364 | 34.482 | 9.657 | Present study |
| *Pelophylax saharicus* | T6838 | BEV.T6838 | KY490365 | 36.626 | 4.508 | Present study |
| *Pelophylax saharicus* | BEV.T7903 | BEV.T7903 | KY490373 | 36.457 | 4.107 | Present study |
| *Pelophylax saharicus* | T7904 | BEV.T7904 | KY490366 | 36.457 | 4.107 | Present study |
| *Pelophylax saharicus* | T7906 | BEV.T7906 | KY490367 | 36.875 | 6.432 | Present study |
| *Pelophylax saharicus* | T7908 | BEV.T7908 | KY490368 | 36.366 | 2.692 | Present study |
| *Pelophylax saharicus* | BEV.T8097 | BEV.T8097 | KY490369 | 33.897 | 2.485 | Present study |
| *Pelophylax saharicus* | BEV.T8103 | BEV.T8103 | KY490370 | 36.867 | 8.377 | Present study |
| *Pelophylax saharicus* | BEV.T8104 | BEV.T8104 | KY490371 | 36.895 | 8.522 | Present study |
| *Pelophylax saharicus* | BEV.T8105 | BEV.T8105 | KY490372 | 36.293 | 7.99 | Present study |
| *Pelophylax saharicus* | pel1853 | BEV.T9621 | KY490376 | 34.927 | 7.037 | Present study |
| *Pelophylax saharicus* | pel668 | BEV.T9689 | KY490377 | 36.034 | 4.158 | Present study |
| *Pelophylax saharicus* | pel669 | BEV.T9690 | KY490378 | 36.034 | 4.158 | Present study |
| *Pelophylax saharicus* | T9702 | BEV.T9702 | KY490361 | 36.827 | 7.922 | Present study |
| *Discoglossus pictus* | BEV.10111 | BEV.10111 | MH142689 | 35.655 | -0.881 | Present study |
| *Discoglossus pictus* | BEV.11328 | BEV.11328 | MH142693 | 34.068 | -2.978 | Present study |
| *Discoglossus pictus* | BEV.11329 | BEV.11329 | MH142694 | 34.068 | -2.978 | Present study |
| *Discoglossus pictus* | BEV.11393 | BEV.11393 | MH142683 | 36.89 | 15.07 | Present study |
| *Discoglossus pictus* | BEV.11396 | BEV.11396 | MH142672 | 37.94 | 12.84 | Present study |
| *Discoglossus pictus* | BEV.11921 | BEV.11921 | MH142696 | 33.961 | -3.044 | Present study |
| *Discoglossus pictus* | BEV.12649 | BEV.12649 | MH142641 | 36.479 | 4.308 | Present study |
| *Discoglossus pictus* | BEV.12650 | BEV.12650 | MH142642 | 36.629 | 4.505 | Present study |
| *Discoglossus pictus* | BEV.13219 | BEV.13219 | MH142655 | 36.743 | 4.319 | Present study |
| *Discoglossus pictus* | BEV.13220 | BEV.13220 | MH142656 | 36.743 | 4.319 | Present study |
| *Discoglossus pictus* | BEV.13240 | BEV.13240 | MH142657 | 36.752 | 4.37 | Present study |
| *Discoglossus pictus* | BEV.13241 | BEV.13241 | MH142658 | 36.752 | 4.37 | Present study |
| *Discoglossus pictus* | BEV.13257 | BEV.13257 | MH142659 | 36.457 | 4.107 | Present study |
| *Discoglossus pictus* | BEV.13258 | BEV.13258 | MH142660 | 36.457 | 4.107 | Present study |
| *Discoglossus pictus* | BEV.13267 | BEV.13267 | MH142661 | 36.761 | 4.435 | Present study |
| *Discoglossus pictus* | BEV.13268 | BEV.13268 | MH142662 | 36.761 | 4.435 | Present study |
| *Discoglossus pictus* | BEV.13279 | BEV.13279 | MH142663 | 35.841 | 2.025 | Present study |
| *Discoglossus pictus* | BEV.13280 | BEV.13280 | MH142664 | 35.456 | 1.537 | Present study |
| *Discoglossus pictus* | BEV.13337 | BEV.13337 | MH142665 | 36.78 | 4.43 | Present study |
| *Discoglossus pictus* | BEV.13338 | BEV.13338 | MH142666 | 36.785 | 4.558 | Present study |
| *Discoglossus pictus* | BEV.13351 | BEV.13351 | MH142667 | 36.544 | 5.567 | Present study |
| *Discoglossus pictus* | BEV.13352 | BEV.13352 | MH142668 | 36.875 | 6.432 | Present study |
| *Discoglossus pictus* | BEV.13361 | BEV.13361 | MH142669 | 36.774 | 5.629 | Present study |
| *Discoglossus pictus* | BEV.13705 | BEV.13705 | MH142679 | 35.748 | -0.575 | Present study |
| *Discoglossus pictus* | BEV.13706 | BEV.13706 | MH142681 | 35.748 | -0.575 | Present study |
| *Discoglossus pictus* | BEV.13707 | BEV.13707 | MH142682 | 35.324 | 6.754 | Present study |
| *Discoglossus pictus* | BEV.13708 | BEV.13708 | MH142674 | 36.793 | 8.374 | Present study |
| *Discoglossus pictus* | BEV.13735 | BEV.13735 | MH142680 | 35.862 | 5.025 | Present study |
| *Discoglossus pictus* | BEV.13736 | BEV.13736 | MH142675 | 36.828 | 7.916 | Present study |
| *Discoglossus pictus* | BEV.13737 | BEV.13737 | MH142673 | 35.234 | -0.722 | Present study |
| *Discoglossus pictus* | BEV.6974 | BEV.6974 | MH142695 | 36.956 | 7.777 | Present study |
| *Discoglossus pictus* | BEV.8778 | BEV.8778 | MH142684 | 38.06 | 12.83 | Present study |
| *Discoglossus pictus* | BEV.8779 | BEV.8779 | MH142671 | 38.06 | 12.83 | Present study |
| *Discoglossus pictus* | BEV.9192 | BEV.9192 | MH142685 | 36.723 | 4.609 | Present study |
| *Discoglossus pictus* | BEV.9213 | BEV.9213 | MH142686 | 36.812 | 5.919 | Present study |
| *Discoglossus pictus* | BEV.9214 | BEV.9214 | MH142687 | 36.812 | 5.919 | Present study |
| *Discoglossus pictus* | BEV.9215 | BEV.9215 | MH142688 | 36.812 | 5.919 | Present study |
| *Discoglossus pictus* | BEV.9217 | BEV.9217 | MH142690 | 36.812 | 5.919 | Present study |
| *Discoglossus pictus* | BEV.9218 | BEV.9218 | MH142691 | 36.812 | 5.919 | Present study |
| *Discoglossus pictus* | BEV.9236 | BEV.9236 | MH142692 | 35.335 | -0.008 | Present study |
| *Discoglossus pictus* | BEV.T6840 | BEV.T6840 | MH142643 | 36.508 | 4.28 | Present study |
| *Discoglossus pictus* | BEV.T7058 | BEV.T7058 | MH142644 | 35.174 | 8.827 | Present study |
| *Discoglossus pictus* | BEV.T7059 | BEV.T7059 | MH142645 | 35.174 | 8.827 | Present study |
| *Discoglossus pictus* | BEV.T7060 | BEV.T7060 | MH142646 | 35.667 | 10.099 | Present study |
| *Discoglossus pictus* | BEV.T7061 | BEV.T7061 | MH142647 | 35.667 | 10.099 | Present study |
| *Discoglossus pictus* | BEV.T7063 | BEV.T7063 | MH142648 | 35.759 | 10.814 | Present study |
| *Discoglossus pictus* | BEV.T7064 | BEV.T7064 | MH142649 | 35.759 | 10.814 | Present study |
| *Discoglossus pictus* | BEV.T7068 | BEV.T7068 | MH142650 | 36.643 | 9.217 | Present study |
| *Discoglossus pictus* | BEV.T7069 | BEV.T7069 | MH142651 | 36.643 | 9.217 | Present study |
| *Discoglossus pictus* | BEV.T7070 | BEV.T7070 | MH142652 | 36.858 | 8.353 | Present study |
| *Discoglossus pictus* | BEV.T7071 | BEV.T7071 | MH142653 | 36.858 | 8.353 | Present study |
| *Discoglossus pictus* | BEV.T7076 | BEV.T7076 | MH142654 | 36.892 | 8.308 | Present study |
| *Discoglossus pictus* | BEV.T7100 | BEV.T7100 | MH142670 | 35.283 | -0.758 | Present study |
| *Discoglossus pictus* | BEV.T8052 | BEV.T8052 | MH142678 | 35.865 | 1.968 | Present study |
| *Discoglossus pictus* | BEV.T9700 | BEV.T9700 | MH142676 | 36.925 | 7.669 | Present study |
| *Discoglossus pictus* | BEV.T9723 | BEV.T9723 | MH142677 | 35.654 | -0.977 | Present study |
| *Acanthodactylus savignyi* | savigyni | BEV.T627 | KR050123 | 35.752 | -0.828 | Miralles et al. unpublished |
| *Acanthodactylus margaritae* | busacki | T4282 | KR049951 | 29.801 | -9.81 | Miralles et al. unpublished |
| *Acanthodactylus erythrurus* | ESSA10 | BEV.11610 | KR049954 | 31.455 | -9.756 | Miralles et al. unpublished |
| *Acanthodactylus erythrurus* | ESSA11 | BEV.11603 | KR049955 | 31.455 | -9.756 | Miralles et al. unpublished |
| *Acanthodactylus erythrurus* | HEBR1 | BEV.11946 | KR049984 | 33.382 | -5.132 | Miralles et al. unpublished |
| *Acanthodactylus erythrurus* | IFRA1 | BEV.11925 | KR049985 | 33.583 | -4.935 | Miralles et al. unpublished |
| *Acanthodactylus erythrurus* | IFRA10 | BEV.11938 | KR049986 | 33.573 | -4.932 | Miralles et al. unpublished |
| *Acanthodactylus erythrurus* | IFRA11 | BEV.11939 | KR049987 | 33.573 | -4.932 | Miralles et al. unpublished |
| *Acanthodactylus erythrurus* | IZEL1 | BEV.12006 | KR050003 | 32.218 | -5.55 | Miralles et al. unpublished |
| *Acanthodactylus erythrurus* | IZEL10 | BEV.11997 | KR050004 | 32.218 | -5.55 | Miralles et al. unpublished |
| *Acanthodactylus erythrurus* | IZEL11 | BEV.11996 | KR050005 | 32.218 | -5.55 | Miralles et al. unpublished |
| *Acanthodactylus erythrurus* | IZEL2 | BEV.12005 | KR050006 | 32.218 | -5.55 | Miralles et al. unpublished |
| *Acanthodactylus erythrurus* | KETA1 | BEV.11897 | KR050014 | 34.937 | -4.616 | Miralles et al. unpublished |
| *Acanthodactylus erythrurus* | LARA1 | BEV.11249 | KR050029 | 35.209 | -6.142 | Miralles et al. unpublished |
| *Acanthodactylus erythrurus* | LARA10 | BEV.11882 | KR050030 | 35.209 | -6.142 | Miralles et al. unpublished |
| *Acanthodactylus erythrurus* | LARA11 | BEV.11883 | KR050031 | 35.209 | -6.142 | Miralles et al. unpublished |
| *Acanthodactylus erythrurus* | MARA1 | BEV.11579 | KR050050 | 31.818 | -7.978 | Miralles et al. unpublished |
| *Acanthodactylus erythrurus* | MARA10 | BEV.11808 | KR050051 | 31.792 | -7.981 | Miralles et al. unpublished |
| *Acanthodactylus erythrurus* | MART1 | BEV.11871 | KR050063 | 35.638 | -5.278 | Miralles et al. unpublished |
| *Acanthodactylus erythrurus* | MART2 | BEV.11872 | KR050064 | 35.638 | -5.278 | Miralles et al. unpublished |
| *Acanthodactylus erythrurus* | MBOU1 | A482 | KR050069 | 34.896 | -6.288 | Miralles et al. unpublished |
| *Acanthodactylus erythrurus* | MBOU10 | A491 | KR050070 | 34.896 | -6.288 | Miralles et al. unpublished |
| *Acanthodactylus erythrurus* | MEHD1 | BEV.11557 | KR050084 | 34.249 | -6.68 | Miralles et al. unpublished |
| *Acanthodactylus erythrurus* | MEHD10 | BEV.11895 | KR050085 | 34.249 | -6.68 | Miralles et al. unpublished |
| *Acanthodactylus erythrurus* | MEHD2 | BEV.11558 | KR050086 | 34.249 | -6.68 | Miralles et al. unpublished |
| *Acanthodactylus erythrurus* | MEHD3 | BEV.11556 | KR050087 | 34.249 | -6.68 | Miralles et al. unpublished |
| *Acanthodactylus erythrurus* | OGHA2 | BEV.11632 | KR050094 | 32.879 | -8.859 | Miralles et al. unpublished |
| *Acanthodactylus erythrurus* | OGHA3 | BEV.11631 | KR050095 | 32.879 | -8.859 | Miralles et al. unpublished |
| *Acanthodactylus erythrurus* | OGHA4 | BEV.11633 | KR050097 | 32.879 | -8.859 | Miralles et al. unpublished |
| *Acanthodactylus erythrurus* | OUAO1 | BEV.12011 | KR050101 | 32.294 | -5.659 | Miralles et al. unpublished |
| *Acanthodactylus erythrurus* | OULM1 | BEV.11230 | KR050102 | 33.45 | -6.081 | Miralles et al. unpublished |
| *Acanthodactylus erythrurus* | OULM10 | BEV.11236 | KR050103 | 33.45 | -6.081 | Miralles et al. unpublished |
| *Acanthodactylus erythrurus* | PORT | BEV.10458 | KR050122 | 38.137 | -8.787 | Miralles et al. unpublished |
| *Acanthodactylus erythrurus* | SBOU1 | BEV.11638 | KR050124 |  |  | Miralles et al. unpublished |
| *Acanthodactylus erythrurus* | SBOU10 | BEV.11644 | KR050125 | 33.38 | -8.226 | Miralles et al. unpublished |
| *Acanthodactylus erythrurus* | SBOU2 | BEV.11639 | KR050126 | 33.38 | -8.226 | Miralles et al. unpublished |
| *Acanthodactylus erythrurus* | SDCH1 | BEV.11191 | KR050134 | 31.797 | -8.522 | Miralles et al. unpublished |
| *Acanthodactylus erythrurus* | SDCH2 | BEV.11192 | KR050135 | 31.797 | -8.522 | Miralles et al. unpublished |
| *Acanthodactylus erythrurus* | SDCH3 | BEV.11193 | KR050136 | 31.797 | -8.522 | Miralles et al. unpublished |
| *Acanthodactylus erythrurus* | SDCH4 | BEV.11202 | KR050137 | 31.797 | -8.522 | Miralles et al. unpublished |
| *Acanthodactylus erythrurus* | SIRUA1 | A541 | KR050143 | 30.747 | -7.609 | Miralles et al. unpublished |
| *Acanthodactylus erythrurus* | SIRUA10 | BEV.10459 | KR050144 | 30.747 | -7.609 | Miralles et al. unpublished |
| *Acanthodactylus erythrurus* | SIRUA2 | A542 | KR050145 | 30.747 | -7.609 | Miralles et al. unpublished |
| *Acanthodactylus erythrurus* | SKEM1 | BEV.11253 | KR050152 | 35.279 | -6.066 | Miralles et al. unpublished |
| *Acanthodactylus erythrurus* | SKEM11 | BEV.11263 | KR050153 | 35.279 | -6.066 | Miralles et al. unpublished |
| *Acanthodactylus erythrurus* | SKEM12 | BEV.11279 | KR050154 | 35.279 | -6.066 | Miralles et al. unpublished |
| *Acanthodactylus erythrurus* | SKTN1 | BEV.11265 | KR050177 | 35.397 | -5.966 | Miralles et al. unpublished |
| *Acanthodactylus erythrurus* | SKTN2 | BEV.11266 | KR050178 | 35.397 | -5.966 | Miralles et al. unpublished |
| *Acanthodactylus erythrurus* | SOUI1 | BEV.11173 | KR050179 | 32.032 | -9.341 | Miralles et al. unpublished |
| *Acanthodactylus erythrurus* | SOUI10 | BEV.11621 | KR050180 | 32.032 | -9.341 | Miralles et al. unpublished |
| *Acanthodactylus erythrurus* | SOUI11 | BEV.11615 | KR050181 | 32.032 | -9.341 | Miralles et al. unpublished |
| *Acanthodactylus erythrurus* | SYAH1 | BEV.11647 | KR050201 | 33.855 | -6.913 | Miralles et al. unpublished |
| *Acanthodactylus erythrurus* | SYAH10 | BEV.11652 | KR050202 | 33.855 | -6.913 | Miralles et al. unpublished |
| *Acanthodactylus erythrurus* | SYAH11 | BEV.11657 | KR050203 | 33.855 | -6.913 | Miralles et al. unpublished |
| *Acanthodactylus erythrurus* | TAZA1 | BEV.11830 | KR050225 | 35.09 | -5.16 | Miralles et al. unpublished |
| *Acanthodactylus erythrurus* | TAZA10 | A497 | KR050226 | 34.104 | -4.072 | Miralles et al. unpublished |
| *Acanthodactylus erythrurus* | TAZA11 | A815 | KR050227 | 34.071 | -4.181 | Miralles et al. unpublished |
| *Acanthodactylus erythrurus* | TIMT1 | BEV.11595 | KR050236 | 31.088 | -9.139 | Miralles et al. unpublished |
| *Acanthodactylus erythrurus* | TIMT2 | BEV.11596 | KR050237 | 31.088 | -9.139 | Miralles et al. unpublished |
| *Acanthodactylus erythrurus* | TIMT3 | BEV.11822 | KR050238 | 31.088 | -9.139 | Miralles et al. unpublished |
| *Acanthodactylus erythrurus* | TISL1 | BEV.11995 | KR050239 | 32.203 | -5.637 | Miralles et al. unpublished |
| *Acanthodactylus erythrurus* | TISL10 | BEV.11985 | KR050240 | 32.203 | -5.637 | Miralles et al. unpublished |
| *Acanthodactylus erythrurus* | TISL11 | BEV.11984 | KR050241 | 32.203 | -5.637 | Miralles et al. unpublished |
| *Acanthodactylus erythrurus* | TITI1 | BEV.11800 | KR050253 | 31.288 | -7.382 | Miralles et al. unpublished |
| *Acanthodactylus erythrurus* | TITI2 | BEV.11799 | KR050254 | 31.288 | -7.382 | Miralles et al. unpublished |
| *Acanthodactylus erythrurus* | TITI3 | BEV.11798 | KR050255 | 31.288 | -7.382 | Miralles et al. unpublished |
| *Chalcides ocellatus* | EU278168 |  | EU278168 | 34.881 | -2.428 | [1] |
| *Chalcides ocellatus* | EU278166 |  | EU278166 | 35.133 | -2.412 | [1] |
| *Chalcides ocellatus* | EU278164 |  | EU278164 | 34.583 | -2.51 | [1] |
| *Chalcides ocellatus* | EU278163 |  | EU278163 | 35.109 | -2.362 | [1] |
| *Chalcides ocellatus* | EU278167 |  | EU278167 | 35.133 | -2.412 | [1] |
| *Chalcides ocellatus* | EU278197 |  | EU278197 | 34.664 | 11.241 | [1] |
| *Chalcides ocellatus* | EU278196 |  | EU278196 | 34.7 | 11.181 | [1] |
| *Chalcides ocellatus* | EU278195 |  | EU278195 | 34.7 | 11.181 | [1] |
| *Chalcides ocellatus* | EU278194 |  | EU278194 | 34.7 | 11.181 | [1] |
| *Chalcides ocellatus* | EU278190 |  | EU278190 | 36.748 | 8.496 | [1] |
| *Chalcides ocellatus* | EU278189 |  | EU278189 | 36.561 | 10.595 | [1] |
| *Chalcides ocellatus* | EU278188 |  | EU278188 |  |  | [1] |
| *Chalcides ocellatus* | EU278169 |  | EU278169 | 36.751 | 3.044 | [1] |
| *Chalcides ocellatus* | JQ344284. |  | JQ344284. | 35.863 | 10.61 | [1] |
| *Chalcides ocellatus* | JQ344285 |  | JQ344285 | 36.673 | 8.702 | [1] |
| *Chalcides ocellatus* | F980216 |  | F980216 |  |  | [1] |
| *Eumeces schneideri* | JQ344289 | MVZ:234475 | JQ344289 |  |  | [2] |
| *Hemorrhois nummifer* | AY376742 |  | AY376742 |  |  | [3] |
| *Hemorrhois algirus* | AY486911 |  | AY486911 |  |  | [3] |
| *Hemorhois hippocrepis* | AY486916.1 |  | AY486916.1 |  |  | [3] |
| *Hemorrhois ravergieri* | AY486920 |  | AY486920 |  |  | [3] |
| *Hemorhois hippocrepis* | AY643391 |  | AY643391 |  |  | [4] |
| *Hemorhois hippocrepis* | AY643392 |  | AY643392 |  |  | [4] |
| *Hemorhois hippocrepis* | DQ451952.1 |  | DQ451952.1 | 35.161 | -5.263 | [4] |
| *Hemorhois hippocrepis* | DQ451953.1 |  | DQ451953.1 | 35.82 | -5.646 | [4] |
| *Hemorhois hippocrepis* | DQ451954.1 |  | DQ451954.1 | 35.576 | -5.951 | [4] |
| *Hemorhois hippocrepis* | DQ451955.1 |  | DQ451955.1 | 36.366 | -5.227 | [4] |
| *Hemorhois hippocrepis* | DQ451956.1 |  | DQ451956.1 | 37.922 | -2.986 | [4] |
| *Hemorhois hippocrepis* | DQ451957.1 |  | DQ451957.1 | 38.381 | -6.809 | [4] |
| *Hemorhois hippocrepis* | DQ451958.1 |  | DQ451958.1 | 37.338 | -6.538 | [4] |
| *Hemorhois hippocrepis* | DQ451959.1 |  | DQ451959.1 | 35.576 | -5.951 | [4] |
| *Hemorhois hippocrepis* | DQ451960.1 |  | DQ451960.1 | 37.267 | -7.024 | [4] |
| *Hemorhois hippocrepis* | DQ451961.1 |  | DQ451961.1 | 36.366 | -5.227 | [4] |
| *Hemorhois hippocrepis* | DQ451962.1 |  | DQ451962.1 | 37.611 | -6.167 | [4] |
| *Hemorhois hippocrepis* | DQ451963.1 |  | DQ451963.1 | 35.13 | -2.416 | [4] |
| *Hemorhois hippocrepis* | DQ451964.1 |  | DQ451964.1 | 35.306 | -5.63 | [4] |
| *Hemorhois hippocrepis* | DQ451965.1 |  | DQ451965.1 | 35.471 | -5.406 | [4] |
| *Hemorhois hippocrepis* | DQ451966.1 |  | DQ451966.1 | 35.389 | -5.894 | [4] |
| *Hemorhois hippocrepis* | DQ451967.1 |  | DQ451967.1 | 37.389 | -5.984 | [4] |
| *Hemorhois hippocrepis* | DQ451968.1 |  | DQ451968.1 | 37.84 | -3.352 | [4] |
| *Hemorhois hippocrepis* | DQ451969.1 |  | DQ451969.1 | 35.143 | -4.134 | [4] |
| *Hemorhois hippocrepis* | DQ451970.1 |  | DQ451970.1 | 34.367 | -5.219 | [4] |
| *Hemorhois hippocrepis* | DQ451971.1 |  | DQ451971.1 | 36.757 | 2.846 | [4] |
| *Hemorhois hippocrepis* | DQ451972.1 |  | DQ451972.1 | 35.861 | 4.724 | [4] |
| *Hemorhois hippocrepis* | DQ451973.1 |  | DQ451973.1 | 34.892 | -1.316 | [4] |
| *Hemorhois hippocrepis* | DQ451974.1 |  | DQ451974.1 | 35.589 | -5.363 | [4] |
| *Hemorhois hippocrepis* | DQ451975.1 |  | DQ451975.1 | 31.629 | -7.981 | [4] |
| *Hemorhois hippocrepis* | DQ451976.1 |  | DQ451976.1 | 41.935 | 0.857 | [4] |
| *Hemorhois hippocrepis* | DQ451977.1 |  | DQ451977.1 | 42.167 | 0.895 | [4] |
| *Hemorhois hippocrepis* | DQ451978.1 |  | DQ451978.1 | 41.642 | 2.743 | [4] |
| *Hemorhois hippocrepis* | DQ451979.1 |  | DQ451979.1 | 37.359 | -1.658 | [4] |
| *Hemorhois hippocrepis* | DQ451980.1 |  | DQ451980.1 | 36.777 | -3.733 | [4] |
| *Hemorhois hippocrepis* | DQ451981.1 |  | DQ451981.1 | 36.366 | -5.227 | [4] |
| *Hemorhois hippocrepis* | DQ451982.1 |  | DQ451982.1 | 37.183 | -4.334 | [4] |
| *Hemorhois hippocrepis* | DQ451983.1 |  | DQ451983.1 | 40.213 | -6.464 | [4] |
| *Hemorhois hippocrepis* | DQ451984.1 |  | DQ451984.1 | 33.441 | -5.232 | [4] |
| *Hemorhois hippocrepis* | DQ451985.1 |  | DQ451985.1 | 36.455 | 10.715 | [4] |
| *Hemorhois hippocrepis* | DQ451986.1 |  | DQ451986.1 | 36.772 | 8.605 | [4] |
| *Hemorhois hippocrepis* | DQ451987.1 |  | DQ451987.1 | 36.455 | 10.715 | [4] |
| *Hemorhois hippocrepis* | KP036573.1 |  | KP036573.1 | 39.702 | 3.436 | [4] |
| *Hyla meridionalis* | DQ996427 |  | DQ996427 | 36.95 | 8.766 | [5] |
| *Hyla meridionalis* | DQ996426 |  | DQ996426 | 36.95 | 8.766 | [5] |
| *Hyla meridionalis* | DQ996425 |  | DQ996425 | 36.95 | 8.766 | [5] |
| *Hyla meridionalis* | DQ996424 |  | DQ996424 | 36.744 | 10.917 | [5] |
| *Hyla meridionalis* | DQ996423 |  | DQ996423 | 30.573 | -7.203 | [6] |
| *Hyla meridionalis* | DQ996422 |  | DQ996422 | 30.016 | -9.651 | [6] |
| *Hyla meridionalis* | DQ996421 |  | DQ996421 | 30.154 | -8.576 | [6] |
| *Hyla meridionalis* | DQ996420 |  | DQ996420 | 30.154 | -8.576 | [6] |
| *Hyla meridionalis* | DQ996419 |  | DQ996419 | 30.154 | -8.576 | [6] |
| *Hyla meridionalis* | DQ996418 |  | DQ996418 | 30.154 | -8.576 | [6] |
| *Hyla meridionalis* | DQ996417 |  | DQ996417 | 31.208 | -7.851 | [6] |
| *Hyla meridionalis* | DQ996416 |  | DQ996416 | 36.013 | -5.602 | [6] |
| *Hyla meridionalis* | DQ996415 |  | DQ996415 | 37.127 | -6.48 | [6] |
| *Hyla meridionalis* | DQ996414 |  | DQ996414 | 37.127 | -6.48 | [6] |
| *Hyla meridionalis* | DQ996413 |  | DQ996413 | 37.127 | -6.48 | [6] |
| *Hyla meridionalis* | DQ996412 |  | DQ996412 | 39.704 | -6.482 | [6] |
| *Hyla meridionalis* | DQ996410 |  | DQ996410 | 32.929 | -5.052 | [6] |
| *Hyla meridionalis* | DQ996411 |  | DQ996411 | 32.929 | -5.052 | [6] |
| *Hyla meridionalis* | DQ996409 |  | DQ996409 | 34.994 | -5.918 | [6] |
| *Hyla meridionalis* | DQ996408 |  | DQ996408 | 35.164 | -5.264 | [6] |
| *Hyla meridionalis* | DQ996407 |  | DQ996407 | 34.994 | -5.918 | [6] |
| *Hyla meridionalis* | DQ996406 |  | DQ996406 | 35.164 | -5.264 | [6] |
| *Hyla meridionalis* | DQ996405 |  | DQ996405 | 35.164 | -5.264 | [6] |
| *Hyla meridionalis* | DQ996404 |  | DQ996404 | 35.164 | -5.264 | [6] |
| *Hyla meridionalis* | DQ996403 |  | DQ996403 | 35.458 | -6.033 | [6] |
| *Hyla meridionalis* | DQ996402 |  | DQ996402 | 35.729 | -5.828 | [6] |
| *Hyla meridionalis* | DQ996401 |  | DQ996401 | 35.164 | -5.264 | [6] |
| *Hyla meridionalis* | DQ996400 |  | DQ996400 | 35.164 | -5.264 | [6] |
| *Hyla meridionalis* | FJ226842 |  | FJ226842 | 31.208 | -7.851 | [5] |
| *Hyla meridionalis* | FJ226841 |  | FJ226841 | 43.52 | 4.7 | [5] |
| *Hyla meridionalis* | FJ226840 |  | FJ226840 | 43.91 | 7.84 | [5] |
| *Hyla meridionalis* | FJ226808 |  | FJ226808 | 28.4 | -16.53 | [5] |
| *Hyla meridionalis* | FJ226807 |  | FJ226807 | 28.4 | -16.53 | [5] |
| *Hyla meridionalis* | FJ226806 |  | FJ226806 | 36.3 | -5.72 | [5] |
| *Hyla meridionalis* | FJ226805 |  | FJ226805 | 36.165 | -5.582 | [5] |
| *Hyla meridionalis* | FJ226804 |  | FJ226804 | 37.31 | -8.596 | [5] |
| *Hyla meridionalis* | FJ226803 |  | FJ226803 | 37.31 | -8.596 | [5] |
| *Hyla meridionalis* | FJ226802 |  | FJ226802 | 37.31 | -8.596 | [5] |
| *Hyla meridionalis* | FJ226801 |  | FJ226801 | 35.466 | -6.033 | [5] |
| *Hyla meridionalis* | FJ226800 |  | FJ226800 | 32.683 | -4.75 | [5] |
| *Hyla meridionalis* | FJ226799 |  | FJ226799 | 35.842 | -5.56 | [5] |
| *Hyla meridionalis* | FJ226798 |  | FJ226798 | 35.113 | -5.288 | [5] |
| *Hyla meridionalis* | FJ226797 |  | FJ226797 | 43.91 | 7.84 | [5] |
| *Hyla meridionalis* | FJ226796 |  | FJ226796 | 43.91 | 7.84 | [5] |
| *Hyla meridionalis* | FJ226795 |  | FJ226795 | 43.91 | 7.84 | [5] |
| *Hyla meridionalis* | DQ996429 |  | DQ996429 | 36.744 | 10.917 | [5] |
| *Hyla meridionalis* | DQ996428 |  | DQ996428 | 36.744 | 10.917 | [5] |
| *Hyla arborea* | Harbore1 |  | FJ226835 |  |  | [5] |
| *Hyla arborea* | Harbore2 |  | FJ226836 |  |  | [5] |
| *Hyla arborea* | Harbore3 |  | FJ226837 |  |  | [5] |
| *Hyla savignyi* | Hsavign2 |  |  |  |  | [5] |
|  |  |  | FJ226849 |  |  |  |
|  |  |  |  |  |  |  |
| *Hyla chinensis* | AY458593 |  | AY458593 |  |  | [7] |
| *Natrix maura* | AY487785 |  | AY487785 |  |  | [8] |
| *Natrix maura* | AY487786 |  | AY487786 | 44.478 | 5.027 | [8] |
| *Natrix maura* | AY873706 |  | AY873706 | 44.999 | 9.017 | [8] |
| *Natrix maura* | AY873707 |  | AY873707 |  |  | [8] |
| *Natrix maura* | AY873708 |  | AY873708 | 36.567 | -6.174 | [8] |
| *Natrix maura* | AY873709 |  | AY873709 | 35.81 | -5.563 | [8] |
| *Natrix maura* | EU437551 |  | EU437551 | 33.924 | -6.485 | [9] |
| *Natrix maura* | EU437552 |  | EU437552 | 30.713 | -7.618 | [8] |
| *Natrix maura* | EU437553 |  | EU437553 | 30.713 | -7.618 | [8] |
| *Natrix maura* | EU437554 |  | EU437554 | 30.713 | -7.618 | [8] |
| *Natrix maura* | EU437555 |  | EU437555 | 34.518 | -4.634 | [8] |
| *Natrix maura* | EU437556 |  | EU437556 | 35.119 | -2.341 | [8] |
| *Natrix maura* | EU437557 |  | EU437557 | 35.119 | -2.341 | [8] |
| *Natrix maura* | EU437558 |  | EU437558 | 35.119 | -2.341 | [8] |
| *Natrix maura* | EU437559 |  | EU437559 | 35.574 | 8.672 | [8] |
| *Natrix maura* | EU437560 |  | EU437560 | 36.47 | -4.991 | [8] |
| *Natrix maura* | EU437561 |  | EU437561 | 36.47 | -4.991 | [8] |
| *Natrix maura* | EU437562 |  | EU437562 | 31.205 | -7.855 | [8] |
| *Natrix maura* | EU437563 |  | EU437563 | 35.573 | 8.668 | [8] |
| *Natrix maura* | EU437564 |  | EU437564 | 36.813 | 9.266 | [8] |
| *Natrix maura* | EU437565 |  | EU437565 | 34.808 | -5.355 | [8] |
| *Natrix maura* | EU437566 |  | EU437566 | 34.915 | -4.572 | [8] |
| *Natrix maura* | EU437567 |  | EU437567 | 33.533 | -5.002 | [8] |
| *Natrix maura* | EU437568 |  | EU437568 | 36.191 | 4.395 | [8] |
| *Natrix maura* | EU437569 |  | EU437569 | 35.58 | 6.076 | [8] |
| *Natrix maura* | EU437570 |  | EU437570 | 35.207 | 6.302 | [8] |
| *Natrix maura* | EU437571 |  | EU437571 | 35.542 | 4.003 | [8] |
| *Natrix maura* | EU437572 |  | EU437572 | 37.002 | -7.884 | [8] |
| *Natrix maura* | KC570222 |  | KC570222 |  |  | [10] |
| *Natrix maura* | KC570223 |  | KC570223 |  |  | [10] |
| *Natrix maura* | KC570224 |  | KC570224 |  |  | [10] |
| *Natrix maura* | KC570225 |  | KC570225 |  |  | [9] |
| *Natrix maura* | KC570226 |  | KC570226 |  |  | [9] |
| *Natrix maura* | KC570227 |  | KC570227 |  |  | [9] |
| *Natrix maura* | KC570228 |  | KC570228 |  |  | [9] |
| *Natrix maura* | KC570229 |  | KC570229 |  |  | [9] |
| *Natrix maura* | KC570230 |  | KC570230 |  |  | [9] |
| *Natrix maura* | KC570231 |  | KC570231 |  |  | [9] |
| *Natrix maura* | KC570232 |  | KC570232 |  |  | [9] |
| *Pelophylax saharicus* | KP177787 |  | KP177787 | 34.818 | -6.302 | [11] |
| *Pelophylax saharicus* | KP177788 |  | KP177788 | 34.818 | -6.302 | [10] |
| *Pelophylax saharicus* |  |  |  | 34.888 | -2.299 | [10] |
| *Pelophylax saharicus* | KP177704 |  | KP177704 | 34.888 | -2.299 | [10] |
| *Pelophylax saharicus* | KP177680 |  | KP177680 | 28.996 | -10.53 | [10] |
| *Pelophylax saharicus* | KP177681 |  | KP177681 | 28.996 | -10.53 | [10] |
| *Pelophylax saharicus* | KP177682 |  | KP177682 | 28.996 | -10.53 | [10] |
| *Pelophylax saharicus* | KP177683 |  | KP177683 | 28.996 | -10.53 | [10] |
| *Pelophylax saharicus* | KP177684 |  | KP177684 | 28.996 | -10.53 | [10] |
| *Pelophylax saharicus* |  |  |  | 31.63 | -8.248 | [10] |
| *Pelophylax saharicus* | KP177673 |  | KP177673 | 29.082 | -10.27 | [11] |
| *Pelophylax saharicus* | KP177674 |  | KP177674 | 29.082 | -10.27 | [11] |
| *Pelophylax saharicus* | KP177675 |  | KP177675 | 29.082 | -10.27 | [11] |
| *Pelophylax saharicus* | KP177676 |  | KP177676 | 29.082 | -10.27 | [11] |
| *Pelophylax saharicus* | KP177677 |  | KP177677 | 29.082 | -10.27 | [11] |
| *Pelophylax saharicus* | KP177762 |  | KP177762 | 35.238 | -5.175 | [11] |
| *Pelophylax saharicus* | KP177763 |  | KP177763 | 35.238 | -5.175 | [11] |
| *Pelophylax saharicus* | KP177764 |  | KP177764 | 35.238 | -5.175 | [11] |
| *Pelophylax saharicus* | KP177765 |  | KP177765 | 35.238 | -5.175 | [11] |
| *Pelophylax saharicus* | KP177697 |  | KP177697 | 33.396 | -5.911 | [11] |
| *Pelophylax saharicus* | KP177698 |  | KP177698 | 33.396 | -5.911 | [11] |
| *Pelophylax saharicus* | KP177699 |  | KP177699 | 33.396 | -5.911 | [11] |
| *Pelophylax saharicus* | KP177700 |  | KP177700 | 33.396 | -5.911 | [11] |
| *Pelophylax saharicus* | KP177701 |  | KP177701 | 33.396 | -5.911 | [11] |
| *Pelophylax saharicus* | KP177702 |  | KP177702 | 33.396 | -5.911 | [11] |
| *Pelophylax saharicus* | KP177703 |  | KP177703 | 33.396 | -5.911 | [11] |
| *Pelophylax saharicus* | KP177692 |  | KP177692 | 31.614 | -9.581 | [11] |
| *Pelophylax saharicus* | KP177693 |  | KP177693 | 31.614 | -9.581 | [11] |
| *Pelophylax saharicus* | KP177718 |  | KP177718 | 35.089 | -5.155 | [11] |
| *Pelophylax saharicus* | KP177719 |  | KP177719 | 35.089 | -5.155 | [11] |
| *Pelophylax saharicus* | KP177720 |  | KP177720 | 35.089 | -5.155 | [11] |
| *Pelophylax saharicus* | KP177727 |  | KP177727 | 35.089 | -5.155 | [11] |
| *Pelophylax saharicus* |  |  |  | 35.089 | -5.155 | [11] |
| *Pelophylax saharicus* | KP177728 |  | KP177728 | 35.089 | -5.155 | [11] |
| *Pelophylax saharicus* | KP177729 |  | KP177729 | 35.089 | -5.155 | [11] |
| *Pelophylax saharicus* | KP177730 |  | KP177730 | 35.089 | -5.155 | [11] |
| *Pelophylax saharicus* | KP177731 |  | KP177731 | 35.089 | -5.155 | [11] |
| *Pelophylax saharicus* | KP177732 |  | KP177732 | 35.089 | -5.155 | [11] |
| *Pelophylax saharicus* | KP177733 |  | KP177733 | 35.089 | -5.155 | [11] |
| *Pelophylax saharicus* | KP177734 |  | KP177734 | 35.089 | -5.155 | [11] |
| *Pelophylax saharicus* | KP177735 |  | KP177735 | 35.089 | -5.155 | [11] |
| *Pelophylax saharicus* | KP177759 |  | KP177759 | 35.254 | -5.216 | [11] |
| *Pelophylax saharicus* | KP177760 |  | KP177760 | 35.254 | -5.216 | [11] |
| *Pelophylax saharicus* | KP177761 |  | KP177761 | 35.254 | -5.216 | [11] |
| *Pelophylax saharicus* | KP177696 |  | KP177696 | 31.771 | -8.708 | [11] |
| *Pelophylax saharicus* | KP177678 |  | KP177678 | 35.005 | -5.68 | [11] |
| *Pelophylax saharicus* | KP177679 |  | KP177679 | 35.005 | -5.68 | [11] |
| *Pelophylax saharicus* | HM363036 |  | HM363036 | 35.15 | 8.53 | [12] |
| *Pelophylax saharicus* | HM363037 |  | HM363037 | 35.15 | 8.53 | [11] |
| *Pelophylax saharicus* | HM363038 |  | HM363038 | 35.15 | 8.53 | [11] |
| *Pelophylax saharicus* | HM363039 |  | HM363039 | 35.15 | 8.53 | [11] |
| *Pelophylax saharicus* |  |  |  | 37.26 | 9.87 | [11] |
| *Pelophylax saharicus* | HM363036 |  | HM363036 | 33.86 | 10.6 | [11] |
| *Pelophylax saharicus* | HM363040 |  | HM363040 | 33.86 | 10.6 | [11] |
| *Pelophylax saharicus* | HM363044 |  | HM363044 | 33.86 | 10.6 | [11] |
| *Pelophylax saharicus* | HM363041 |  | HM363041 | 34.41 | 8.78 | [11] |
| *Pelophylax saharicus* | HM363041 |  | HM363041 | 34.41 | 8.78 | [11] |
| *Pelophylax saharicus* | HM363043 |  | HM363043 | 36.43 | 8.41 | [11] |
| *Pelophylax saharicus* | HM363043 |  | HM363043 | 36.43 | 8.41 | [11] |
| *Pelophylax saharicus* |  |  |  | 37.15 | 9.65 | [11] |
| *Pelophylax saharicus* | HM363042 |  | HM363042 | 35.66 | 10.8 | [11] |
| *Pelophylax saharicus* | HM363037 |  | HM363037 | 35.16 | 8.81 | [11] |
| *Pelophylax saharicus* | HM363037 |  | HM363037 | 35.16 | 8.81 | [11] |
| *Pelophylax saharicus* | HM363033 |  | HM363033 | 33.75 | 8.82 | [11] |
| *Pelophylax saharicus* | HM363036 |  | HM363036 | 33.75 | 8.82 | [11] |
| *Pelophylax saharicus* | HM363036 |  | HM363036 | 36.84 | 11.1 | [11] |
| *Pelophylax saharicus* | HM363036 |  | HM363036 | 36.84 | 11.1 | [11] |
| *Pelophylax saharicus* | HM363036 |  | HM363036 | 35.75 | 10.8 | [11] |
| *Pelophylax saharicus* | HM363036 |  | HM363036 | 35.75 | 10.8 | [11] |
| *Pelophylax saharicus* | HM363030 |  | HM363030 | 36.95 | 8.86 | [11] |
| *Pelophylax saharicus* | HM363031 |  | HM363031 | 36.95 | 8.86 | [11] |
| *Pelophylax saharicus* | HM363031 |  | HM363031 | 36.64 | 9.21 | [11] |
| *Pelophylax saharicus* | HM363036 |  | HM363036 | 36.64 | 9.21 | [11] |
| *Pelophylax saharicus* | HM363034 |  | HM363034 | 34.38 | 7.6 | [11] |
| *Pelophylax saharicus* | HM363035 |  | HM363035 | 34.38 | 7.6 | [11] |
| *Pelophylax saharicus* | HM363032 |  | HM363032 | 36.86 | 10.16 | [11] |
| *Pelophylax saharicus* | HM363033 |  | HM363033 | 36.86 | 10.16 | [11] |
| *Rana huanrensis* | KT588071 |  | KT588071 |  |  | [13] |
| *Podarcis vaucheri* | EF081082 |  | EF081082 |  |  | [14] |
| *Podarcis vaucheri* | EF081094 |  | EF081094 |  |  | [13] |
| *Podarcis vaucheri* | EF081099 |  | EF081099 |  |  | [13] |
| *Podarcis vaucheri* | EF081111 |  | EF081111 |  |  | [13] |
| *Podarcis vaucheri* | EF081114 |  | EF081114 |  |  | [13] |
| *Podarcis vaucheri* | EF081115 |  | EF081115 |  |  | [13] |
| *Podarcis vaucheri* | HQ898024 |  | HQ898024 |  |  | [15] |
| *Podarcis vaucheri* | HQ898027 |  | HQ898027 |  |  | [15] |
| *Podarcis vaucheri* | HQ898028 |  | HQ898028 |  |  | [15] |
| *Podarcis vaucheri* | HQ898029 |  | HQ898029 |  |  | [15] |
| *Podarcis vaucheri* | DQ081177 |  | DQ081177 |  |  | [16] |
| *Podarcis vaucheri* | DQ081178 |  | DQ081178 |  |  | [16] |
| *Podarcis vaucheri* | HQ898013 |  | HQ898013 |  |  | [14] |
| *Podarcis vaucheri* | HQ898012 |  | HQ898012 |  |  | [14] |
| *Podarcis vaucheri* | HQ898030 |  | HQ898030 |  |  | [14] |
| *Podarcis vaucheri* | HQ898031 |  | HQ898031 |  |  | [14] |
| *Podarcis vaucheri* | GQ856102 |  | GQ856102 |  |  | [14] |
| *Podarcis vaucheri* | GQ856106 |  | GQ856106 |  |  | [14] |
| *Podarcis vaucheri* | EU269587 |  | EU269587 |  |  | [17] |
| *Podarcis vaucheri* | GQ856108 |  | GQ856108 |  |  | [18] |
| *Podarcis vaucheri* | GQ856104 |  | GQ856104 |  |  | [18] |
| *Podarcis vaucheri* | GQ856105 |  | GQ856105 |  |  | [18] |
| *Podarcis vaucheri* | GQ856103 |  | GQ856103 |  |  | [18] |
| *Podarcis vaucheri* | GQ856100 |  | GQ856100 |  |  | [16] |
| *Podarcis vaucheri* | DQ081180 |  | DQ081180 |  |  | [16] |
| *Podarcis vaucheri* | HQ898009 |  | HQ898009 |  |  | [15] |
| *Podarcis vaucheri* | GQ856101 |  | GQ856101 |  |  | [18] |
| *Podarcis vaucheri* | EU269585 |  | EU269585 |  |  | [16] |
| *Podarcis vaucheri* | DQ081179 |  | DQ081179 |  |  | [16] |
| *Podarcis vaucheri* | EU269568 |  | EU269568 |  |  | [16] |
| *Podarcis vaucheri* | DQ081170 |  | DQ081170 |  |  | [16] |
| *Podarcis vaucheri* | GQ856098 |  | GQ856098 |  |  | [13] |
| *Podarcis vaucheri* | GQ856099 |  | GQ856099 |  |  | [13] |
| *Podarcis vaucheri* | GQ856096 |  | GQ856096 |  |  | [13] |
| *Podarcis vaucheri* | GQ856097 |  | GQ856097 |  |  | [13] |
| *Podarcis vaucheri* | GQ856094 |  | GQ856094 |  |  | [13] |
| *Podarcis vaucheri* | GQ856095 |  | GQ856095 |  |  | [13] |
| *Ptyodactylus oudrii (12S rRNA)* | 1 |  | GU195714 |  |  | [19] |
| *Ptyodactylus oudrii (12S rRNA)* | 2 |  | GU195715 |  |  | [18] |
| *Ptyodactylus oudrii (12S rRNA)* | 3 |  | GU195716 |  |  | [18] |
| *Ptyodactylus oudrii (12S rRNA)* | 4 |  | GU195717 |  |  | [18] |
| *Ptyodactylus oudrii (12S rRNA)* | 5 |  | GU195718 |  |  | [18] |
| *Ptyodactylus oudrii (12S rRNA)* | 6 |  | GU195719 |  |  | [18] |
| *Ptyodactylus oudrii (12S rRNA)* | 7 |  | GU195720 |  |  | [18] |
| *Ptyodactylus oudrii (12S rRNA)* | 8 |  | GU195721 |  |  | [18] |
| *Ptyodactylus oudrii (12S rRNA)* | 9 |  | GU195722 |  |  | [18] |
| *Ptyodactylus oudrii (12S rRNA)* | 10 |  | GU195723 |  |  | [18] |
| *Ptyodactylus oudrii (12S rRNA)* | 11 |  | GU195724 |  |  | [18] |
| *Ptyodactylus oudrii (12S rRNA)* | 12 |  | GU195725 |  |  | [18] |
| *Ptyodactylus oudrii (12S rRNA)* | 13 |  | GU195726 |  |  | [18] |
| *Ptyodactylus oudrii (12S rRNA)* | 14 |  | GU195727 |  |  | [18] |
| *Ptyodactylus oudrii (12S rRNA)* | 15 |  | GU195728 |  |  | [18] |
| *Ptyodactylus oudrii (12S rRNA)* | 16 |  | GU195729 |  |  | [18] |
| *Ptyodactylus oudrii (12S rRNA)* | 17 |  | GU195730 |  |  | [18] |
| *Ptyodactylus oudrii (12S rRNA)* | 18 |  | GU195731 |  |  | [18] |
| *Ptyodactylus oudrii (12S rRNA)* | 19 |  | GU195732 |  |  | [18] |
| *Ptyodactylus oudrii (12S rRNA)* | 20 |  | GU195737 |  |  | [18] |
| *Ptyodactylus oudrii (12S rRNA)* | 21 |  | GU195738 |  |  | [18] |
| *Ptyodactylus oudrii (12S rRNA)* | 22 |  | GU195739 |  |  | [18] |
| *Ptyodactylus oudrii (12S rRNA)* | 23 |  | GU195740 |  |  | [18] |
| *Ptyodactylus oudrii (12S rRNA)* | 24 |  | GU195741 |  |  | [18] |
| *Ptyodactylus oudrii (12S rRNA)* | 25 |  | GU195742 |  |  | [18] |
| *Ptyodactylus oudrii (12S rRNA)* | 26 |  | GU195743 |  |  | [18] |
| *Ptyodactylus oudrii (12S rRNA)* | 27 |  | GU195744 |  |  | [18] |
| *Ptyodactylus oudrii (12S rRNA)* | 28 |  | GU195745 |  |  | [18] |
| *Ptyodactylus oudrii (12S rRNA)* | 29 |  | GU195746 |  |  | [18] |
| *Ptyodactylus oudrii (12S rRNA)* | 30 |  | GU195735 |  |  | [18] |
| *Ptyodactylus oudrii (12S rRNA)* | 31 |  | GU195736 |  |  | [18] |
| *Ptyodactylus siphonorhina (12S rRNA)* | 32 |  | GU195733 |  |  | [18] |
| *Ptyodactylus togoensis (12S rRNA)* | 33 |  | GU195734 |  |  | [18] |
| *Ptyodactylus togoensis (12S rRNA)* | 34 |  | GU195747 |  |  | [18] |
| *Ptyodactylus togoensis (16S rRNA)* | 1 |  | GU195748 |  |  | [18] |
| *Ptyodactylus togoensis (16S rRNA)* | 2 |  | GU195749 |  |  | [18] |
| *Ptyodactylus togoensis (16S rRNA)* | 3 |  | GU195750 |  |  | [18] |
| *Ptyodactylus togoensis (16S rRNA)* | 4 |  | GU195751 |  |  | [18] |
| *Ptyodactylus togoensis (16S rRNA)* | 5 |  | GU195752 |  |  | [18] |
| *Ptyodactylus togoensis (16S rRNA)* | 6 |  | GU195753 |  |  | [18] |
| *Ptyodactylus togoensis (16S rRNA)* | 7 |  | GU195754 |  |  | [18] |
| *Ptyodactylus togoensis (16S rRNA)* | 8 |  | GU195755 |  |  | [18] |
| *Ptyodactylus togoensis (16S rRNA)* | 9 |  | GU195756 |  |  | [18] |
| *Ptyodactylus togoensis (16S rRNA)* | 10 |  | GU195757 |  |  | [18] |
| *Ptyodactylus togoensis (16S rRNA)* | 11 |  | GU195758 |  |  | [18] |
| *Ptyodactylus togoensis (16S rRNA)* | 12 |  | GU195759 |  |  | [18] |
| *Ptyodactylus togoensis (16S rRNA)* | 13 |  | GU195760 |  |  | [18] |
| *Ptyodactylus togoensis (16S rRNA)* | 14 |  | GU195761 |  |  | [18] |
| *Ptyodactylus togoensis (16S rRNA)* | 15 |  | GU195762 |  |  | [18] |
| *Ptyodactylus togoensis (16S rRNA)* | 16 |  | GU195763 |  |  | [18] |
| *Ptyodactylus togoensis (16S rRNA)* | 17 |  | GU195764 |  |  | [18] |
| *Ptyodactylus togoensis (16S rRNA)* | 18 |  | GU195765 |  |  | [18] |
| *Ptyodactylus togoensis (16S rRNA)* | 19 |  | GU195766 |  |  | [18] |
| *Ptyodactylus togoensis (16S rRNA)* | 20 |  | GU195771 |  |  | [18] |
| *Ptyodactylus togoensis (16S rRNA)* | 21 |  | GU195772 |  |  | [18] |
| *Ptyodactylus togoensis (16S rRNA)* | 22 |  | GU195773 |  |  | [18] |
| *Ptyodactylus togoensis (16S rRNA)* | 23 |  | GU195774 |  |  | [18] |
| *Ptyodactylus togoensis (16S rRNA)* | 24 |  | GU195775 |  |  | [18] |
| *Ptyodactylus togoensis (16S rRNA)* | 25 |  | GU195776 |  |  | [18] |
| *Ptyodactylus togoensis (16S rRNA)* | 26 |  | GU195777 |  |  | [18] |
| *Ptyodactylus togoensis (16S rRNA)* | 27 |  | GU195778 |  |  | [18] |
| *Ptyodactylus togoensis (16S rRNA)* | 28 |  | GU195779 |  |  | [18] |
| *Ptyodactylus togoensis (16S rRNA)* | 29 |  | GU195780 |  |  | [18] |
| *Ptyodactylushaselquistii* | 30 |  | GU195769 |  |  | [18] |
| *Ptyodactylushaselquistii* | 31 |  | GU195770 |  |  | [18] |
| *Ptyodactylus siphonorhina (12S rRNA)* | 32 |  | GU195767 |  |  | [18] |
| *Ptyodactylus togoensis (12S rRNA)* | 33 |  | GU195768 |  |  | [18] |
| *Ptyodactylus togoensis (12S rRNA)* | 34 |  | GU195781 |  |  | [18] |
| *Ptyodactylus oudrii* | KP858152 |  |  |  |  | [20] |
| *Ptyodactylus oudrii* | KP858344 |  |  |  |  | [19] |
| *Ptyodactylus oudrii* | KP858415 |  |  |  |  | [19] |
| *Ptyodactylus oudrii* | KP858192 |  |  |  |  | [19] |
| *Ptyodactylus oudrii* | KP858450 |  |  |  |  | [19] |
| *Ptyodactylus oudrii* | KP858451 |  |  |  |  | [19] |
| *Ptyodactylus oudrii* | KP858470 |  |  |  |  | [19] |
| *Ptyodactylus oudrii* | KP858232 |  |  |  |  | [19] |
| *Ptyodactylus oudrii* | KP858428 |  |  |  |  | [19] |
| *Ptyodactylus oudrii* | KP858429 |  |  |  |  | [19] |
| *Ptyodactylus oudrii* | KP858148 |  |  |  |  | [19] |
| *Ptyodactylus oudrii* | KP858149 |  |  |  |  | [19] |
| *Ptyodactylus togoensis* | KC505531 |  |  |  |  | [19] |
| *Ptyodactylus togoensis* | KC505532 |  |  |  |  | [19] |
| *Ptyodactylus togoensis* | KC505533 |  |  |  |  | [19] |
| *Ptyodactylus togoensis* | KC505534 |  |  |  |  | [19] |
| *Timon tangitanus* | HQ287269 |  |  |  |  | [21] |
| *Timon tangitanus* | HQ287267 |  |  |  |  | [20] |
| *Timon tangitanus* | HQ287266 |  |  |  |  | [20] |
| *Timon tangitanus* | HQ287265 |  |  |  |  | [20] |
| *Timon tangitanus* | HQ287264 |  |  |  |  | [20] |
| *Timon tangitanus* | HQ287263 |  |  |  |  | [20] |
| *Timon tangitanus* | HQ287261 |  |  |  |  | [20] |
| *Timon tangitanus* | HQ287262 |  |  |  |  | [20] |
| *Timon tangitanus* | HQ287260 |  |  |  |  | [20] |
| *Timon tangitanus* | HQ287259 |  |  |  |  | [20] |
| *Timon tangitanus* | HQ287258 |  |  |  |  | [20] |
| *Timon tangitanus* | HQ287257 |  |  |  |  | [20] |
| *Timon tangitanus* | HQ287256 |  |  |  |  | [20] |
| *Timon tangitanus* | HQ287255 |  |  |  |  | [20] |
| *Timon tangitanus* | HQ287254 |  |  |  |  | [20] |
| *Timon tangitanus* | HQ287253 |  |  |  |  | [20] |
| *Timon tangitanus* | HQ287252 |  |  |  |  | [20] |
| *Timon tangitanus* | HQ287251 |  |  |  |  | [20] |
| *Timon tangitanus* | HQ287250 |  |  |  |  | [20] |
| *Timon tangitanus* | HQ287249 |  |  |  |  | [20] |
| *Timon tangitanus* | HQ287248 |  |  |  |  | [20] |
| *Timon tangitanus* | HQ287247 |  |  |  |  | [20] |
| *Timon tangitanus* | HQ287246 |  |  |  |  | [20] |
| *Timon tangitanus* | HQ287245 |  |  |  |  | [20] |
| *Timon tangitanus* | HQ287244 |  |  |  |  | [20] |
| *Timon tangitanus* | HQ287268 |  |  |  |  | [20] |
| *Timon tangitanus* | AF378956 |  |  |  |  | [22] |
| *Timon pater* | AF378958 |  |  |  |  | [22] |
| *Timon tangitanus* | AF378955 |  |  |  |  | [22] |
| *Trogonophis wiegmanni* | TR1r |  | EF545727 |  |  | [23] |
| *Trogonophis wiegmanni* | TR2r |  | EF545729 |  |  | [22] |
| *Trogonophis wiegmanni* | TR2 |  | EF545713 |  |  | [22] |
| *Trogonophis wiegmanni* | TR3 |  | EF545715 |  |  | [22] |
| *Trogonophis wiegmanni* | TR4 |  | EF545717 |  |  | [22] |
| *Trogonophis wiegmanni* | TR5 |  | EF545720 |  |  | [22] |
| *Trogonophis wiegmanni* | TR6 |  | EF545721 |  |  | [22] |
| *Trogonophis wiegmanni* | TR6_2 |  | EF545723 |  |  | [22] |
| *Trogonophis wiegmanni* | TR7_2 |  | EF545725 |  |  | [22] |
| *Trogonophis wiegmanni* | TR7_68 |  | EF545731 |  |  | [22] |
| *Trogonophis wiegmanni* | TR7_61 |  | EF545733 |  |  | [22] |
| *Trogonophis wiegmanni* | TR1_39r |  | EF545735 |  |  | [22] |
| *Trogonophis wiegmanni* | EU203659 |  | EU203659 |  |  | [22] |
| *Trogonophis wiegmanni* | MVZ162544 |  | MVZ162544 |  |  | [22] |
| *Trogonophis wiegmanni* | T26 |  | KJ624789 |  |  | [22] |
| *Trogonophis wiegmanni (12S rRNA)* | TR1r |  | EF545726 |  |  | [22] |
| *Trogonophis wiegmanni (12S rRNA)* | TR2r |  | EF545728 |  |  | [22] |
| *Trogonophis wiegmanni (12S rRNA)* | TR2 |  | EF545712 |  |  | [22] |
| *Trogonophis wiegmanni (12S rRNA)* | TR3 |  | EF545714 |  |  | [22] |
| *Trogonophis wiegmanni (12S rRNA)* | TR4 |  | EF545716 |  |  | [22] |
| *Trogonophis wiegmanni (12S rRNA)* | TR5 |  | EF545718 |  |  | [22] |
| *Trogonophis wiegmanni (12S rRNA)* | TR6 |  | EF545720 |  |  | [22] |
| *Trogonophis wiegmanni (12S rRNA)* | TR6_2 |  | EF545722 |  |  | [22] |
| *Trogonophis wiegmanni (12S rRNA)* | TR7_2 |  | EF545724 |  |  | [22] |
| *Trogonophis wiegmanni (12S rRNA)* | TR7_68 |  | EF545730 |  |  | [22] |
| *Trogonophis wiegmanni (12S rRNA)* | TR7_61 |  | EF545732 |  |  | [22] |
| *Trogonophis wiegmanni (12S rRNA)* | TR1_39r |  | EF545734 |  |  | [22] |
| *Discoglossus scovazzi* | KF644929S |  | KF644929 |  |  | [24] |
| *Discoglossus scovazzi* | KF644927S |  | KF644927 |  |  | [24] |
| *Discoglossus scovazzi* | KF644928S |  | KF644928 |  |  | [24] |
| *Discoglossus scovazzi* | KF644926S |  | KF644926 |  |  | [24] |
| *Discoglossus jeanneae* | DQ902149J |  | DQ902149 |  |  | [25] |
|  |  |  |  |  |  |  |
| *Blanus cinereus* | cinereus1 |  | EU443257 |  |  | [26] |
| *Blanus cinereus* | Cinereus2 |  | KJ624883 |  |  | [27] |
| *Blanus strauchi* | strauchi |  | KJ624855 |  |  | [27] |
| *Trogonophis wiegmanni (12S rRNA)* | EU203659 |  | EU203659 |  |  | [22] |

**Literature cited in S1 Table**

1. Carranza S, Arnold EN, Geniez P, Roca J, Mateo JA. Radiation, multiple dispersal and parallelism in the skinks, Chalcides and Sphenops (Squamata: Scincidae), with comments on Scincus and Scincopus and the age of the Sahara Desert. Mol Phylogenet Evol. 2008;46: 1071–1094. doi:10.1016/j.ympev.2007.11.018

2. Lavin BR, Papenfuss TJ. The phylogenetic position of Chalcides ocellatus (Squamata: Scincidae) from Yemen and Somalia. 2012;36: 26–36.

3. Nagy ZT, Lawson R, Joger U, Wink M. Molecular systematics of racers, whipsnakes and relatives (Reptilia: Colubridae) using mitochondrial and nuclear markers. J Zool Syst Evol Res. Wiley Online Library; 2004;42: 223–233.

4. Carranza S, Arnold EN, Pleguezuelos JM. Phylogeny, biogeography, and evolution of two Mediterranean snakes, Malpolon monspessulanus and Hemorrhois hippocrepis (Squamata, Colubridae), using mtDNA sequences. Mol Phylogenet Evol. 2006;40: 532–546. doi:10.1016/j.ympev.2006.03.028

5. Stöck M, Dubey S, Klütsch C, Litvinchuk SN, Scheidt U, Perrin N. Mitochondrial and nuclear phylogeny of circum-Mediterranean tree frogs from the Hyla arborea group. Mol Phylogenet Evol. 2008;49: 1019–1024. doi:10.1016/j.ympev.2008.08.029

6. Recuero E, Iraola A, Rubio X, Machordom A, García-París M. Mitochondrial differentiation and biogeography of Hyla meridionalis (Anura: Hylidae): an unusual phylogeographical pattern. J Biogeogr. 2007;34: 1207–1219. doi:10.1111/j.1365-2699.2007.01688.x

7. Zhang P, Zhou H, Chen Y-Q, Liu Y-F, Qu L-H. Mitogenomic perspectives on the origin and phylogeny of living amphibians. Syst Biol. Society of Systematic Zoology; 2005;54: 391–400.

8. Guicking D, Griffiths R a., Moore RD, Joger U, Wink M. Introduced alien or persecuted native? Resolving the origin of the viperine snake (Natrix Maura) on Mallorca. Biodivers Conserv. 2006;15: 3045–3054. doi:10.1007/s10531-005-4878-y

9. Barata M, Harris DJ, Castilho R. Comparative phylogeography of northwest African Natrix maura ( Serpentes : Colubridae ) inferred from mtDNA sequences. African Zool. 2008;43: 1–7. doi:10.3377/1562-7020(2008)43[1:CPONAN]2.0.CO;2

10. Kindler C, Böhme W, Corti C, Gvoždík V, Jablonski D, Jandzik D, et al. Mitochondrial phylogeography, contact zones and taxonomy of grass snakes (Natrix natrix, N. megalocephala). Zool Scr. 2013;42: 458–472. doi:10.1111/zsc.12018

11. Nicolas, V., Mataame, A., Crochet, P. A., Geniez, P., & Ohler, A. (2015). Phylogeographic patterns in north African water frog Pelophylax saharicus (Anura: Ranidae). Journal of Zoological Systematics and Evolutionary Research, 53(3), 239-248.

12. Nabil A, Sarra F, Paolo M, Slim B-Y, Khaled S. Assessment of intraspecific mtDNA variability of the water frog Pelophylax saharicus in Eastern North Africa. Ann Zool. 2010;60: 639–646. doi:10.3161/000345410X550508

13. Dong B, Zhou Y, Yang B. The complete mitochondrial genome of the Rana huanrensis (Anura: Ranidae). Mitochondrial DNA Part A. Taylor & Francis; 2016;27: 4551–4552.

14. Lima A, Pinho C, Larbes S, Carretero MA, Brito JC, Harris DJ. Relationships of Podarcis wall lizards from Algeria based on mtDNA data. Amphibia-Reptilia. Brill; 2009;30: 483–492.

15. Kaliontzopoulou a., Brito JC, Carretero M a., Larbes S, Harris DJ. Modelling the partially unknown distribution of wall lizards (Podarcis) in North Africa: ecological affinities, potential areas of occurrence, and methodological constraints. Can J Zool. 2008;86: 992–1001. doi:10.1139/Z08-078

16. Pinho C, Ferrand N, Harris DJ. Reexamination of the Iberian and North African Podarcis (Squamata: Lacertidae) phylogeny based on increased mitochondrial DNA sequencing. Mol Phylogenet Evol. 2006;38: 266–73. doi:10.1016/j.ympev.2005.06.012

17. Pinho C, Harris DJ, Ferrand N. Comparing patterns of nuclear and mitochondrial divergence in a cryptic species complex: the case of Iberian and North African wall lizards (Podarcis, Lacertidae). Biol J Linn Soc. Wiley Online Library; 2007;91: 121–133.

18. Lima A, Pinho C, Larbes S, Carretero M, Brito JC, Harris DJ. Relationships of Podarcis wall lizards from Algeria based on mtDNA data. Amphibia-Reptilia. 2009;30: 483–492. doi:10.1163/156853809789647103

19. Perera A, Harris DJ, Harris J. Genetic variability within the Oudri ’s fan-footed gecko Ptyodactylus oudrii in North Africa assessed using mitochondrial and nuclear DNA sequences. Mol Phylogenet Evol. 2010;54: 634–639. doi:10.1016/j.ympev.2009.10.020

20. Metallinou M, Červenka J, Crochet P-A, Kratochvíl L, Wilms T, Geniez P, et al. Species on the rocks: Systematics and biogeography of the rock-dwelling Ptyodactylus geckos (Squamata: Phyllodactylidae) in North Africa and Arabia. Mol Phylogenet Evol. 2015;85: 208–220. doi:10.1016/j.ympev.2015.02.010

21. Perera A, Harris DJ. Genetic Variability in the Ocellated Lizard Timon tangitanus in Morocco. African Zool. Zoological Society of Southern Africa; 2010;45: 321–329. doi:10.3377/004.045.0211

22. Paulo OS, Pinheiro J, Miraldo a., Bruford MW, Jordan WC, Nichols R a. The role of vicariance vs. dispersal in shaping genetic patterns in ocellated lizard species in the western Mediterranean. Mol Ecol. 2008;17: 1535–1551. doi:10.1111/j.1365-294X.2008.03706.x

23. Mendonça B, Harris DJ. Genetic variation within Trogonophis wiegmanni Kaup 1830. Belgian J Zool. 2007;137: 239–242.

24. Vences M, De Pous P, Nicolas V, Díaz-Rodríguez J, Donaire D, Hugemann K, et al. New insights on phylogeography and distribution of painted frogs (Discoglossus) in northern Africa and the Iberian Peninsula. Amphibia-Reptilia. Brill; 2014;35: 305–320.

25. Busack SD, Lawson R. Morphological, mitochondrial DNA and allozyme evolution in representative amphibians and reptiles inhabiting each side of the Strait of Gibraltar. Biol J Linn Soc. 2008;94: 445–461. doi:10.1111/j.1095-8312.2008.00992.x

26. Albert EM, San Mauro D, García-París M, Rüber L, Zardoya R. Effect of taxon sampling on recovering the phylogeny of squamate reptiles based on complete mitochondrial genome and nuclear gene sequence data. Gene. Elsevier; 2009;441: 12–21.

27. Sampaio FL, Harris DJ, Perera A, Salvi D. Phylogenetic and diversity patterns of Blanus worm lizards (Squamata: Amphisbaenia): insights from mitochondrial and nuclear gene genealogies and species tree. J Zool Syst Evol Res. Wiley Online Library; 2015;53: 45–54.
