# Supplementary material for: Comparative phylogeography of amphibians and reptiles in Algeria suggests common causes for the east-west phylogeographic breaks in the Maghreb
Source: PLoS One. 2018 Aug 29;13(8):e0201218. doi: 10.1371/journal.pone.0201218 (PMC6114291; doi:10.1371/journal.pone.0201218)
Supplement: S2 Table — (DOCX) [file pone.0201218.s002.docx]

**S2 Table. List of primers, their references and hybridisation temperatures**

| ***Taxon*** | **Gene** | **Primer 5’-3’** | **Reference** | **Hybridization** |
| --- | --- | --- | --- | --- |
| *Acanthodactylus erythrurus*  *Podarcis vaucheri*  *Natrix maura* | ND4 and tRNA | **ND4-F** CACCTATGACTACCAAAAGCTCATGTAGAAGC  **ND4-R** TTACTTTTACTTGGAGTTGCACCA | [1] | 54°C  58°C  56°C |
| *Chalcides ocellatus* | CytB | **CytB2** TCAGAATGATATTTGTCCTCA  **Gludge** TGACTTGAARAACCAYCGTTG | [2]  [3] | 50°C |
| *Discoglossus pictus* | CytB | **CBDL1** ATGGCACCCAACATCCGNAAATC  **CBDL2** TGAGGACAAATATCTTTTTGAGG | Present study | 52°C |
| *Hemorrhois hippocrepis* | CytB | L14724 CGAAGCTTGATATGAAAAACCATCGTTG  H15547b AATAGGAAGTATCATTCTGGTTTAATG | [4] and see [5] | 52°C |
| *Hyla meridionalis*  *Pelophylax saharicus* | CO1 | **Amp3F** CAATACCAAACCCCCTTRTTYGTWTGATC  **Amp3R** GCTTCTCARATAATAAATATYAT | [6] | 42°C  46°C |
| *Timon pater,*  *Timon tangitanus*  *Timon wiegmanni*  *Ptyodactylus oudrii* | 16S RNA | **16SAR-L** CGCCTGTTTATCAAAAACAT  **L02510** CGCCTGTTTATCAAAAACAT | [7] | 51°C  51°C  51°C  52° |

**References cited in S2 table.**

1. Arèvalo E, Davis SK, Sites JW. Mitochondrial DNA Sequence Divergence and Phylogenetic Relationships among Eight Chromosome Races of the Sceloporus Grammicus Complex (Phrynosomatidae) in Central Mexico. Syst Biol . 1994;43: 387–418. doi:10.1093/sysbio/43.3.387

2. Smith MF, Patton, James L. The diversification of South American murid rodents: evidence from mitochondrial DNA sequence data for the akodontine tribe. Biol J Linn Soc. Blackwell Publishing Ltd; 1993;50: 149–177. doi:10.1111/j.1095-8312.1993.tb00924.x

3. McGuire JA, Witt CC, Altshuler DL, Remsen J V. Phylogenetic Systematics and Biogeography of Hummingbirds: Bayesian and Maximum Likelihood Analyses of Partitioned Data and Selection of an Appropriate Partitioning Strategy. Syst Biol . 2007;56: 837–856. doi:10.1080/10635150701656360

4. Irwin DM, Kocher TD, Wilson AC. Evolution of the cytochromeb gene of mammals. J Mol Evol. 1991;32: 128–144. doi:10.1007/BF02515385

5. Kelly CMR, Barker NP, Villet MH. Phylogenetics of advanced snakes (Caenophidia) based on four mitochondrial genes. Syst Biol. 2003;52: 439–459. doi:10.1080/10635150390218132

6. Chiari Y, Vences M, Vieites DR, Rabemananjara F, Bora P, Ramilijaona Ravoahangimalala O, et al. New evidence for parallel evolution of colour patterns in Malagasy poison frogs (Mantella). Mol Ecol. 2004;13: 3763–74. doi:10.1111/j.1365-294X.2004.02367.x

7. Kocher TD, Thomas WK, Meyer a, Edwards S V, Pääbo S, Villablanca FX, et al. Dynamics of mitochondrial DNA evolution in animals: amplification and sequencing with conserved primers. Proc Natl Acad Sci U S A. 1989;86: 6196–6200. doi:10.1073/pnas.86.16.6196
